# Meeting report: GenBank microbial genomic taxonomy workshop (12–13 May, 2015)

**DOI:** 10.1186/s40793-016-0134-1

**Published:** 2016-02-09

**Authors:** Scott Federhen, Ramon Rossello-Mora, Hans-Peter Klenk, Brian J. Tindall, Konstantinos T. Konstantinidis, William B. Whitman, Daniel Brown, David Labeda, David Ussery, George M. Garrity, Rita R. Colwell, Nur Hasan, Joerg Graf, Aidan Parte, Pablo Yarza, Brittany Goldberg, Heike Sichtig, Ilene Karsch-Mizrachi, Karen Clark, Richard McVeigh, Kim D. Pruitt, Tatiana Tatusova, Robert Falk, Seán Turner, Thomas Madden, Paul Kitts, Avi Kimchi, William Klimke, Richa Agarwala, Michael DiCuccio, James Ostell

**Affiliations:** NCBI, Bethesda, USA; IMEDEA (CSIC-UIB), Esporles, Spain; Newcastle University, Newcastle upon Tyne, UK; DSMZ, Braunschweig, Germany; Georgia Institute of Technology, Atlanta, USA; University of Georgia, Athens, USA; University of Florida, Gainesville, USA; USDA, Washington D.C, USA; Oak Ridge National Lab, Oak Ridge, USA; Michigan State University, East Lansing, USA; University of Maryland, College Park, USA; CosmosID, Rockville, USA; University of Connecticut, Storrs, USA; LPSN, New York City, USA; Ribocon, Bremen, Germany; FDA, Silver Spring, USA

**Keywords:** GenBank, Genomic taxonomy, Misidentified sequence entries

## Abstract

Many genomes are incorrectly identified at GenBank. We developed a plan to find and correct misidentified genomes using genomic comparison statistics together with a scaffold of reliably identified genomes from type. A workshop was organized with broad representation from the bacterial taxonomic community to review the proposal, the GenBank Microbial Genomic Taxonomy Workshop, Bethesda MD, May 12–13, 2015.

**Electronic supplementary material:**

The online version of this article (doi:10.1186/s40793-016-0134-1) contains supplementary material, which is available to authorized users.

## Introduction

GenBank serves a dual role as the archive of sequence data for the scientific literature and as the sequence reference database for the research community. Misidentified genomes in GenBank are problems for everyone, and there are many of them – some more egregious than others. In the limited domain of microbial genomes it has recently become possible to reliably find and correct most of these misidentifications using a very simple statistic (average nucleotide identity, ANI) and a scaffold of reliably identified genomes. In the cultured microbes there is a single candidate “reliably identified genome” for each published taxonomic name – the genome from the type strain (subsequently referred to as “type”), which is designated when the name is first described in the taxonomic literature. Recent enhancements to the NCBI Taxonomy database allow us to flag sequences (and genomes) from type [1]. There are currently 4300 genomes from type in GenBank, representing roughly 30 % of the bacterial species with validly published names. For many of the species without genomes from type we have enough sequence from type in GenBank to identify a closely related genome that can serve as a proxy for the type (proxytype analysis, introduced in reference [1] – note that we will not need any proxytype genomes once we have a genome from the type strain of every validly published name). These two datasets (proxytype tables and ANI neighboring tables) are sufficient to find and correct the vast majority of misidentified genomes in GenBank.

This proposal has profound consequences for GenBank and our user communities, and represents a significant change in policy (for a limited domain of sequences). Due to the importance of this decision we convened a workshop for bacterial taxonomists to review the proposal. Here we report the results of that meeting, with a clear mandate to proceed.

## A modest proposal

It has been clear for some time that the evolving data landscape in the bacteria would eventually lead to a transition from DNA-DNA hybridization (DDH) and 16S rRNA sequence-based models of species delimitation to measures based on genome sequences (complete & WGS) from type strains [1–3]. Many genome-wide similarity statistics have emerged including ANI, AAI, dDDH, TETRA, MLSA [4], kmer scores, core- and pan-genome complement and copy number, and others. We focus here on average nucleotide identity (ANI) a simple measure which seems to be entirely adequate for the task of validating species identifications, though we also routinely run kmer, MLSA and core/pan-genome analyses. There are several different ways that ANI has been computed in the literature – we calculate ANI by counting the number of identities across the gapped pairwise alignment between two genomes. This also gives us a measure of the fraction of each genome which contributes to the alignment, as in Varghese et al. 2015 [5] (Table 1).

**Table 1:** Definitions and references for abbreviations and terms used in this paper

Term/abbreviation	Reference	Definition
type	[11]	exemplar strain designated when a new name is described in the taxonomic literature
proxytype	[1]	genome designated (by NCBI) to serve as a proxy for the type, for species that do not yet have a genome from type
genome from type	[1]	genome sequence (complete or WGS) from a type strain
sequence from type	[1]	GenBank sequence from a type strain (generally excluding components of genomes from type)
ANI	[12, 13]	pairwise average nucleotide identity between two genomes, measured across the alignable region.
AAI	[14]	average amino acid identity
dDDH	[15]	digital DNA/DNA hybridization
TETRA	[16]	tetranucleotide frequency distribution
MLSA	[4]	multi-locus sequence analysis. ours is based on ribosomal protein sequences
species complex		a group of closely related species which are difficult to distinguish by traditional methods
suppressed		a sequence entry that has been removed from Entrez, BLAST and the INSDC exchange. It is still retrievable by accession. http://www.insdc.org/documents/insdc-status-document
unverified	[17]	a sequence entry that has been flagged as problematic with the defline token UNVERIFIED. It is indexed in Entrez and exchanged by the INSDC, but is removed from BLAST.
INSDC		International Nucleotide Sequence Database Collaboration, GenBank/ENA/DDBJ http://www.insdc.org/

GenBank has always relied on submitters for the correct taxonomic identification of their sequence entries. Some of these identifications are incorrect; others were correct when they were submitted, but need nomenclature updates due to subsequent taxonomic revisions. GenBank finds and corrects some of the most egregious misidentifications (e.g. bacterial sequences submitted as nematode, or as *Tyrannosaurus rex*) but most species in GenBank are represented by a mere snippet of sequence, and there is no reliable set of reference sequences to anchor the identification.

Two recent developments have changed this situation in the domain of bacterial genomes, allowing GenBank to reliably validate the taxonomic identifications in most submissions. First, sequencing technology has evolved to the point where bacterial genomes are generated cheaply and routinely, and several simple genomic similarity measures have been developed (ANI in particular). Second, the curation of type material in the NCBI taxonomy database has allowed us to flag sequences from type in GenBank [1]. We currently list 4300 genomes from type in the bacteria, roughly 1/3 complete & 2/3 WGS assemblies. This represents about a third of the current species with validly published names. Species lacking genomes from type will often have enough sequence from type in GenBank to designate an appropriate proxy for the type among genomes that we do have. Together these genomes (from type & proxytype) represent a scaffold of reliably identified sequences which we can use to find and correct misidentified genomes in GenBank, and to update the identification of existing entries as new species are described in the literature.

NCBI developed a protocol for using ANI genome neighboring statistics in conjunction with reference genomes from type and proxytype to find misidentified genomes in GenBank. The default rule of thumb ANI cutoff for species boundaries is 96 %, but this is not always appropriate. Many existing species span much more (or much less) that this, so we have added the facility to designate ANI_cutoff values on a species by species basis (see slide 24 in the Additional file 1). In addition, some species are known to be paraphyletic – for example, all four species of *Shigella* fall within the species *Escherichia coli*, which fits snugly within the default 96 % ANI cutoff. Each of the *Shigella* bits can be subtracted out with their own ANI cutoffs from type – 99.2 % works nicely for each of the *Shigella* species in this case (see slide 35 in the Additional file 1).

The analysis which supports the validation of taxonomic identifications relies on two sets of data files – tables of genome neighbors (sorted by ANI) for all genomes from type & proxytype, and tables of proxytype calculations for each bacterial species lacking a genome from type. These will be updated continuously, as new genomes are registered in the NCBI Assembly database, and as new sequences from type are submitted to GenBank. GenBank will designate proxytypes as appropriate, and use the ANI neighboring tables to find and correct misidentified genomes. The type genomes themselves are inviolate, once we establish that the metadata is correct (the sequence is actually from the strain with which it is annotated), that it is correctly assembled and free from contamination. Beyond that, taxonomic issues involving type strains need to be resolved in the taxonomic literature.

For many of the species that do not have genomes from type we can designate a proxy for the type genome. This ‘proxytype analysis’ involves blasting our genomes with whatever shorter sequences from type we have for the species in GenBank – we are looking for the closest genome we have, and are trying to predict where the type genome will fall once we do get it (see slides 11–19, 21 & 31 in the Additional file 1). At the same time this serves to validate the GenBank sequences from type – any entry that is not actually from the type strain will behave differently in this analysis. For example, AF515643.1(1 was submitted as the rpoB sequence from DSM 20477, the type strain of *Enterococcus faecium*, but it did not find a strong hit in the genomes that the other sequences from type for this species preferred, and appeared to have come from a different species (*Serratia grimesii*). We contacted the submitter (who had moved to a different institution in the intervening decade), he consulted his notebooks and replied that he had gotten that strain from a neighboring lab (Luca Cocolin, personal communication). This entry has been suppressed with the approval of the submitter.

**Fig. 1:**
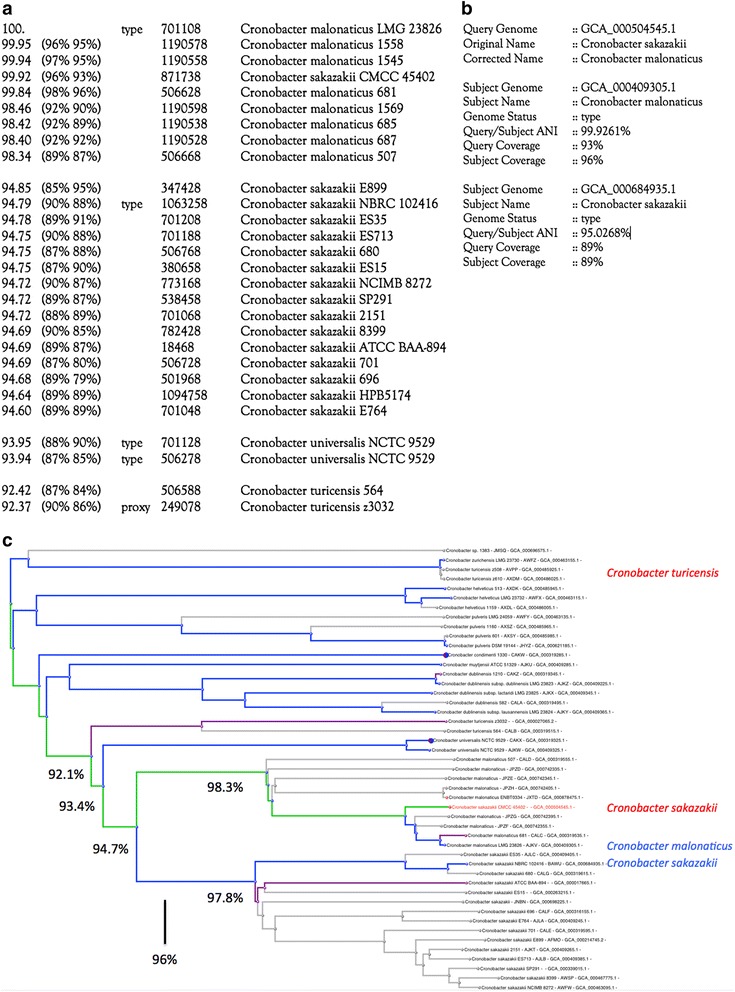
**a** ANI neighboring table for the type genome of *Cronobacter malonaticus*. **b** Structured comment for the GenBank flatfile, summarizing the evidence that supports the taxonomic identification update. **c** Screen shot of the kmer tree showing the misidentified *Cronobacter sakazakii* genome. Type genomes are highlighted in blue, RefSeq reference genomes in purple. ANI spans are shown for several clades. As is evident in Fig. 1a, every genome in the *malonaticus* clade will be very close to 94.7 % ANI with respect to every genome in the *sakazakii* clade. In addition, two misidentified *Cronobacter turicensis* genomes appear at the top of the figure

Figure 1 shows an example of a genome misidentified as *Cronobacter sakazakii* in the ANI neighboring table for the type genome of *Cronobacter malonaticus*, and the proposed structured comment that summarizes the data supporting a change in the identification – the genome in question is 99.9 % identical to the type genome of *Cronobacter malonaticus*, but is only 95.0 % identical to the type genome of *Cronobacter sakazakii*.

GenBank plans to move all of the genome analysis described below to the very front of the submission pipeline so that problems with contamination, identification and classification can be raised directly with the submitters at the time of submission. Issues with existing entries are more problematic – it is often not possible to contact the original submitters; others may be unresponsive, having moved on to different areas of research. There are several alternatives: [1] we could update the RefSeq copy (or simply delete it) and leave the GenBank entry intact [2], we could UNVERIFY (or simply suppress) the GenBank entry, or [3] we could update both the GenBank and RefSeq entries, adding supporting evidence as a comment and notifying submitters of the change by email.

Suppressed entries can be retrieved by accession, but are not indexed in Entrez, exchanged by the INSDC, or found in the BLAST databases. UNVERIFIED entries are indexed in Entrez and exchanged by the INSDC, but are removed from the BLAST databases. Unverifying is often more convenient, since links between sets of sequence entries are maintained – a genome comprised of many pieces can still be retrieved as a set, without having to know all of the constituent accessions.

We believe that the last course or action is best. CP001654 & CP001655 (discussed in reference 1) were unverified last year - CP001654 was submitted as *Dickeya dadantii*, but is 99.99 % identical to the type genome of *Dickeya paradisiaca*, and only 82 % identical to the type genome of *Dickeya dadantii*. CP001655 was submitted as *Dickeya zeae*, but is 96.59 % identical to the type genome of *Dickeya chrysanthemi*, and only 86.9 % identical to the type genome of *Dickeya zeae* (see slides 14–15 in the Additional file 1). Repeated attempts to engage the submitter were fruitless. We believe that it would be more useful to provide a working copy of these genomes with the correct taxonomic identifications, and that it would only cause confusion to maintain a RefSeq genome indexed with a separate name. GenBank has updated the identification of several dozen genomes on an ad hoc basis over the past several months, sending emails to submitters that we plan to update their entries in two weeks unless we hear from them. In most cases we have heard nothing; in the few cases where we have had a response it has always been positive.

GenBank has always exercised some curatorial control over the source features in sequence submissions, most clearly in cases of synonymies that have been established in the taxonomic literature. For example, we list *Homo neanderthalensis* as a synonym of *Homo sapiens neanderthalensis* – we change one name to the other without asking permission of submitters. We also handle subjective synonymies that have been established in the literature – for example, we list *Aeromonas aquariorum* as a synonym of *Aeromonas dhakensis* [6]. The ANI analysis supports this synonymy – the type genome of *Aeromonas aquariorum* is 97.6 % ANI from the type genome of *Aeromonas dhakensis* (see slide 41 in the Additional file 1). The International Nucleotide Sequence Database Collaboration (INSDC) follows the NCBI Taxonomy Database as the international standard nomenclature for sequence annotation.

This proposal extends this control over the organism field in the source feature in a well-defined fashion for a limited set of cases (bacterial genomes) where we are confident that we can correct a misidentification. GenBank will clearly identify entries that we have updated in this fashion, and will include a machine-readable structured comment that summarizes the evidence supporting the update. If the genome is misidentified but is otherwise correctly assembled and free from contamination, we propose to correct the identification in the entry, add an informative machine-readable comment, and notify the submitter of the change. Entries with additional problems will be unverified or suppressed – e.g. entries with contamination, or entries for which the strain identification also appears to be incorrect. We will also provide a mechanism to exempt particular genomes from this analysis if we find convincing evidence of cases for which the method does not work.

These principles may also be applicable to other groups of organisms as well. A kmer distance tree for fungal genomes is already being reviewed at NCBI to uncover potential misidentifications. In particular the unicellular yeasts are largely cultivable species with abundant type strain information, and should be amenable to this analysis as the genome sampling becomes dense enough. The NCBI virus group is also exploring similar methods of genomic taxonomy.

This represents a significant change to GenBank policy, so a workshop with a broad representation of the bacterial taxonomic community was convened to review the proposal. The results of the workshop were presented at the 2015 meeting of the INSDC which was held at the NCBI the week after the taxonomy workshop.

## The workshop

The Microbial Genomic Taxonomy Workshop was held at the NCBI in Bethesda MD on 12–13 May, 2015. The attendees included many officers of the International Committee on Systematics of Prokaryotes (ICSP) and of the Judicial Commission on Prokaryotic Nomenclature, as well as bioinformaticians and taxonomists working with genomic similarity measures. Early discussions with Ramon Rossello-Mora, Hans-Peter Klenk and Brian Tindall indicated that the time was propitious for the workshop, and a limited budget was provided to support travel. Attendees included:

- Ramon Rossello-Mora, IMEDEA (CSIC-UIB)
- Hans-Peter Klenk, Newcastle
- Brian J. Tindall, DSMZ (Chairman, Judicial Commission)
- Pablo Yarza, Ribocon/Silva
- Barny Whitman, UGa. (Treasurer, ICSP)
- Dave Ussery, ORNL/UTenn
- Kostas Kostantinidis, GaTech
- Joerg Graf, UConn
- Dave Labeda, USDA/NRRL (chair, Subcommittee on *Streptomycetaceae*)
- George M. Garrity, MSU/NamesforLife
- Rita Colwell, UMd
- Nur Hasan, UMd
- Daniel Brown, UFl (Secretary of Subcommittees, ICSP)
- Aidan Parte, LPSN.
- Fred Rainey, Iain Sutcliffe & Peter Dawyndt were unable to attend.
- Brittany Goldberg & Heike Sichtig of the FDA phoned in for part of the workshop.

This project spans many groups at the NCBI, including taxonomy (Scott Federhen & Sean Turner), GenBank (Ilene Karsch-Mizrachi), WGS (Karen Clark), SRA (Chris O’Sullivan) 16S rRNA (Rich McVeigh), BLAST (Tom Madden), RefSeq (Kim Pruitt), genomes (Tatiana Tatusov), genome pipeline (Mike DiCuccio), Assembly (Paul Kitts & Avi Kimchi), genome workbench (Bob Falk), genome analysis (Richa Agarwala & Josh Cherry) and the pathogen pipeline (Bill Klimke, Martin Shumway). Jim Ostell, Kim Pruitt and Bill Klimke were out of town and unable to attend. In addition, Conrad Schoch & Barbara Robbertse (representing the fungi), Stacy Ciufo (representing the prokaryotes) and Rodney Brister (representing the viruses) attended as observers – these groups are closely following the developments in bacterial taxonomy with regard to similar efforts in their domains.

There was a single presentation, *A Modest Proposal for making the Genomes of GenBank beneficial to the Publick* (see Additional file 1) which went on for most of the first day of the workshop, interspersed with dynamically scheduled discussion sessions. Tuesday morning was devoted to cementing agreement on the items listed below, and on exploring future directions.

Sequence from type is the foundation of the proposal, and it is essential that this high-value subset of the sequence database is annotated with correct metadata and is free from contamination. Many of the bacterial genomes of GenBank have never been properly screened (by NCBI or by the submitters) for contamination with other bacterial sequence. For example, our type genome for *Mumia flava* was contaminated with *Burkholderia cepacia*, our type genome for *Thauera selenatis* was contaminated with *Enterobacter cloacae*, and our type genome for *Chryseobacterium taeanense* was contaminated with *Delftia* (see slide 42 in the Additional file 1). An improved suite of contamination screens is a necessary precondition for the proposal, and the reference set of type genomes needs to be particularly clean. Thankfully, sequence from type should be internally consistent - the co-identical strains held by different culture collections should all be copies of the same strain and should all have nearly identical genome sequences. As more data accumulates, it is easy to spot sequences which claim to be from type but are not.

We have sequences from type (including both complete and WGS genomes) for ~30 % of the bacterial species with validly published names. For most of the species without genomes from type we have enough sequence from type in GenBank to designate a proxy for the type genome. We spent a considerable amount of time introducing this proxytype analysis and discussing its limitations and applications, in particular its utility in supporting taxonomic inferences in the absence of complete genomes from type. *‘Proxytype’* is an archaic term in plant systematics, used in the early 20th century for what we now call neotypes. Current usage was coined in Federhen (2015) for the process of using sequences from type in GenBank to determine where the type genome is likely to fall once it is sequenced, and to designate a genome that can serve as a proxy for the type until a type genome becomes available [1]. The size of the type sequence set, the strength of the proxytype hits and the local topology of the tree of genomes are all factors which affect the confidence with which we can designate a proxytype genome for any particular species which lacks a genome from type.

We worked through several cases of the protocol in detail, including *Aeromonas* which has been established as something of a test case for this method in the literature ([7, 8] and see slides 37–41 in the Additional file 1)), another look at the *Raoultella*/*Klebsiella* clade which has undergone some considerable changes over the past year (a good indicator of the dynamic nature of microbial taxonomy, slides 16–29 in the Additional file 1) and an analysis of the *Enterobacter cloacae* complex (an explicit attempt to find cases that would challenge the protocol, slides 30–34 in the Additional file 1). The proxytype analysis here makes a strong prediction – that our only genome from *Lelliottia amnigena* is actually an (apparently uncontaminated) genome from *Enterobacter hormaechei* subsp. *steigerwaltii* (aka *Enterobacter**clocae*, *sensu lato*) and that the type genome for *Lelliottia amnigena* would fall somewhere on the long branch leading to the singleton genome identified as *Enterobacter* sp. 638, just outside of the *cloacae* complex. ANI analysis places the type genome for *Lelliottia amnigena* exactly where the proxytype analysis predicts, roughly two-thirds of the way down the long branch leading to *Enterobacter* sp. 638 (Fujita Nobuyuki, unpublished results, and slides 30–32 in the Additional file 1).

Reliable statistics and confidence measures are lacking for most (but not all) of the genome-scaled similarity measures, in comparison with bootstrap values associated with traditional locus-based parsimony and likelihood methods. This is a particularly important area to address. The group also expressed interest in the possibility of providing summary-level statistics on genome quality, contamination screens and taxonomic identification of genome submissions to journal editors and reviewers of the corresponding scientific literature.

Overall, the workshop group was able to reach unanimous agreement on ten items.

- For straightforward cases of misidentification, NCBI should change the name, add informative machine and human-readable comments, and notify the submitter of the update by email.
- On a case by case basis in existing species complexes, NCBI will generate informal names for clades that are not associated with currently published names (by type or proxytype).
- As new species are described NCBI will use ANI (and other measures) to identify clades of existing genomes that need to be updated with names that are consistent with the new nomenclature.
- NCBI will maintain ANI neighboring tables for all of our genomes from type and proxytype, and proxytype tables for all of our sequences from type in GenBank.
- NCBI will maintain ANI_cutoff values on a species-specific basis.
- The question “how identical is co-identical?” should be addressed by the broader community. To support that analysis, NCBI will publish the profile of ANI comparisons between multiple type genomes from the same species. At the start of the project we found 250 species with more than one genome from type. These had pairwise ANI values ranging from 81 % to 100 % (the median was just over 99.99 %)
- NCBI will publish methods for calculating proxytypes, calibration with other measures, and recommendations for their use.
- RefSeq genome quality measures (data and rules) will be published and made public.
- NCBI will move the genome quality analysis, contamination screens, and taxonomic identification validation to the front of the submission pipeline in order to raise and resolve these issues with the submitters as early as possible.
- We strongly recommend that genomes from type are sequenced for every species, and included with new species descriptions. Ideally, multiple genomes from type would be sequenced from copies of type for the same species in different culture collections.

Additional topics included a discussion of ways to keep the type material annotation in the NCBI Taxonomy database current with the literature. The DSM and the LPSN have agreed to share resources in this area, and some information can be parsed out of the Validation and Notification Lists published monthly in the IJSEM. Sadly there is no comprehensive open-access machine-readable validation list of approved names with associated metadata. It also seems advisable to cast a wider net, and examine all of the types collected at StrainInfo [9]. This dataset includes errors inherited from the hundreds of culture collections from which these data were culled, but errors that are associated with sequence data should stand out very clearly and can be identified and corrected or removed.

The workshop group also supported the establishment of a sequencing wishlist as a means to request genome sequences which would be particularly useful for the protocol analysis. One clear example emerged from the discussion – it would be extremely useful to have high-quality genomes from type for every species of bacteria that has been found to be a common contaminant of sequencing reagents. *Delftia acidovorans*, *Delftia tsuruhatensis* and *Burkholderia contaminans* are specific examples that were discussed during the meeting. Of course it would be very useful to have type genomes for all of the taxa that are currently represented only by proxytypes. For example in the *Enterobacter cloacae* complex these include *Enterobacter mori*, *Enterobacter kobei*, *Enterobacter xiangfangensis*, and two subspecies of *Enterobacter hormaechei* whose names are currently effectively, but not validly published –‘*Enterobacter hormaechei* subsp. *steigerwaltii’* & ‘*Enterobacter hormaechei* subsp. *oharae’*. Proxytypes designations for each of these taxa flag unnamed clades in the *cloacae* complex, but we need to validate these assignments with real placements of the type genomes. Several efforts are underway to sequence type strains for all bacterial species, e.g. [10].

The workshop group was also very interested in genomic approaches to the uncultured and currently undescribed components of microbial diversity, and in developing approaches to formalizing nomenclature at different levels in this domain.

And finally, the workshop group agreed to publish a meeting report, and encouraged NCBI to write the methods and analysis paper describing the genomic taxonomy protocol (Federhen et al., In preparation), in addition to publication of the RefSeq genome quality analysis and contamination screening tools.

## Additional file

Additional file 1: Figure S1. A Modest Proposal for making the Genomes of GenBank beneficial to the Publick, and preventing them from being a Burthen to the Curators and Taxonomists. (PDF 9956 kb)
