# Supplementary material for: Meeting report: GenBank microbial genomic taxonomy workshop (12–13 May, 2015)
Source: Stand Genomic Sci. 2016 Feb 9;11:15. doi: 10.1186/s40793-016-0134-1 (PMC4748488; doi:10.1186/s40793-016-0134-1)
Supplement: Additional file 1: Figure S1. — A Modest Proposal for making the Genomes of GenBank beneficial to the Publick, and preventing them from being a Burthen to the Curators and Taxonomists. (PDF 9956 kb) [file 40793_2016_134_MOESM1_ESM.pdf]

A Modest Proposal

for making the *Genomes* of *GenBank* beneficial to the Publick
and preventing them from being a Burthen
to the *Curators* and *Taxonomists*

Apologies to Jonathan Swift for cribbing his title. Talk first given at the NCBI IEB seminar, January 2015. And again at the NCBI Genomic Taxonomy Workshop, 12 May 2015.

About that which we cannot align, we must remain silent.

All things being simple,
every genome within a clade should be statistically equivalent
with respect to every genome outside of the clade.

like **Yi qi** *of the Scansoriopterygidae, a carnivorous flying dinosaur*

three mantras.

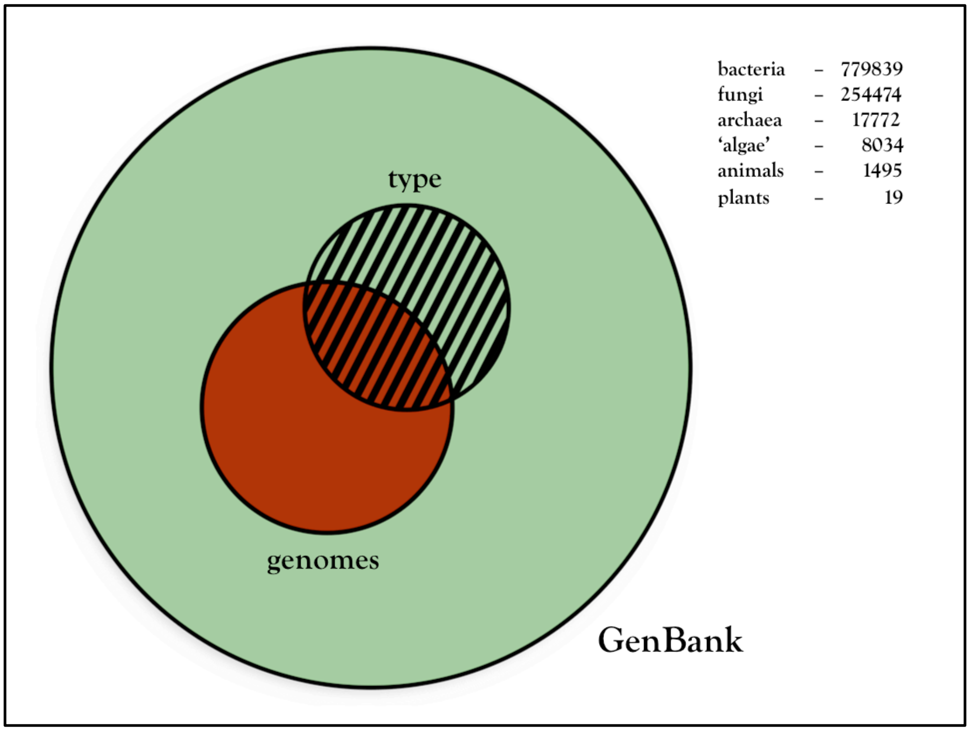

Sequences in GenBank. Some are components of genome submissions & some are taken from type specimens. Some are both & some are neither. Most of our sequences from type are from the cultured microbes, where type material is easily obtained from the culture collections.

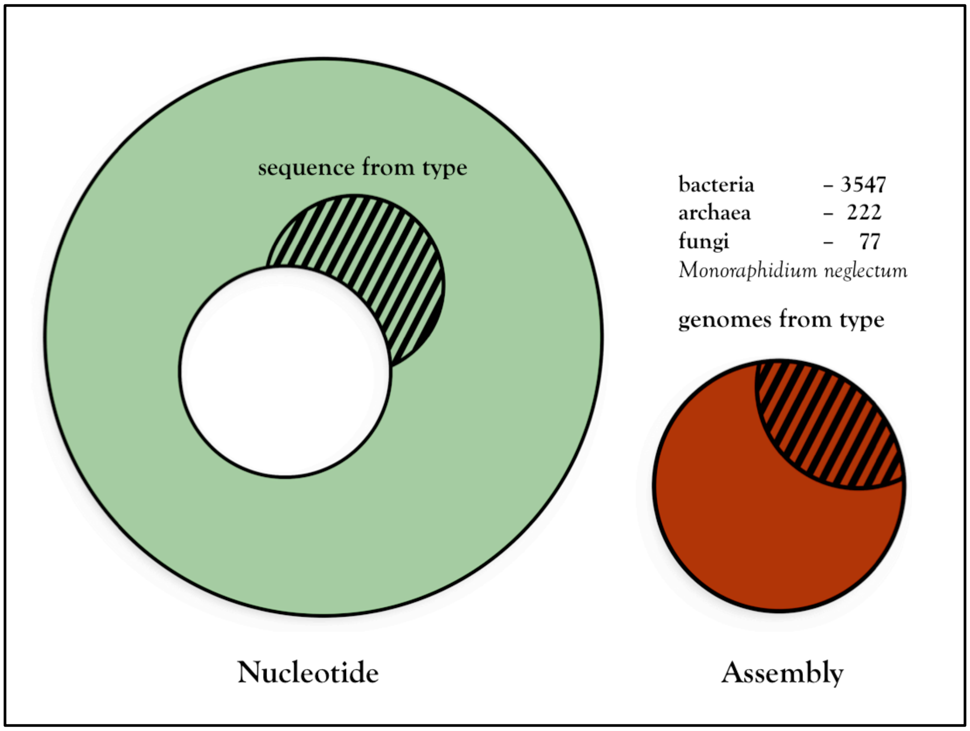

For counting purposes, it is convenient to separate the genome submissions from the rest of GenBank. We have just over 4000 genomes from type, roughly 2/3 from WGS projects and 1/3 complete sequences. The proxytype analysis described below involves blasting the remaining sequence from type in GenBank against all of our genomes (from type and not from type).

100.		type	701108	Cronobacter malonaticus LMG 23826
99.95	(96% 95%)		1190578	Cronobacter malonaticus
99.94	(97% 95%)		1190558	Cronobacter malonaticus
99.92	(96% 93%)		871738	Cronobacter sakazakii CMCC 45402
99.84	(98% 96%)		506628	Cronobacter malonaticus 681
98.46	(92% 90%)		1190598	Cronobacter malonaticus
98.42	(92% 89%)		1190538	Cronobacter malonaticus
98.40	(92% 92%)		1190528	Cronobacter malonaticus
98.34	(89% 87%)		506668	Cronobacter malonaticus 507
94.85	(85% 95%)		347428	Cronobacter sakazakii E899
94.79	(90% 88%)	type	1063258	Cronobacter sakazakii NBRC 102416
94.78	(89% 91%)		701208	Cronobacter sakazakii ES35
94.75	(87% 88%)		506768	Cronobacter sakazakii 680
94.75	(90% 88%)		701188	Cronobacter sakazakii ES713
94.75	(87% 90%)		380658	Cronobacter sakazakii ES15
94.72	(89% 87%)		538458	Cronobacter sakazakii SP291
94.72	(90% 87%)		773168	Cronobacter sakazakii NCIMB 8272
94.72	(88% 89%)		701068	Cronobacter sakazakii 2151
94.69	(87% 80%)		506728	Cronobacter sakazakii 701
94.69	(89% 87%)		18468	Cronobacter sakazakii ATCC BAA-894
94.69	(90% 85%)		782428	Cronobacter sakazakii 8399
94.68	(89% 79%)		501968	Cronobacter sakazakii 696
94.64	(89% 89%)		1094758	Cronobacter sakazakii
94.60	(89% 89%)		701048	Cronobacter sakazakii E764
93.95	(88% 90%)	type	701128	Cronobacter universalis NCTC 9529
93.94	(87% 85%)	type	506278	Cronobacter universalis NCTC 9529
92.42	(87% 84%)		506588	Cronobacter turicensis 564
92.37	(90% 86%)		249078	Cronobacter turicensis z3032

ANI neighboring table for the type genome of Cronobacter malonaticus. Figures in parentheses gives the fraction of each genome which contribute to the pairwise alignment. Note the presence of a putative misidentified genome (assembly id 871738, GenBank CP006731) submitted as Cronobacter sakazakii. Spaces introduced in display to highlight clades with statistically equivalent scores.

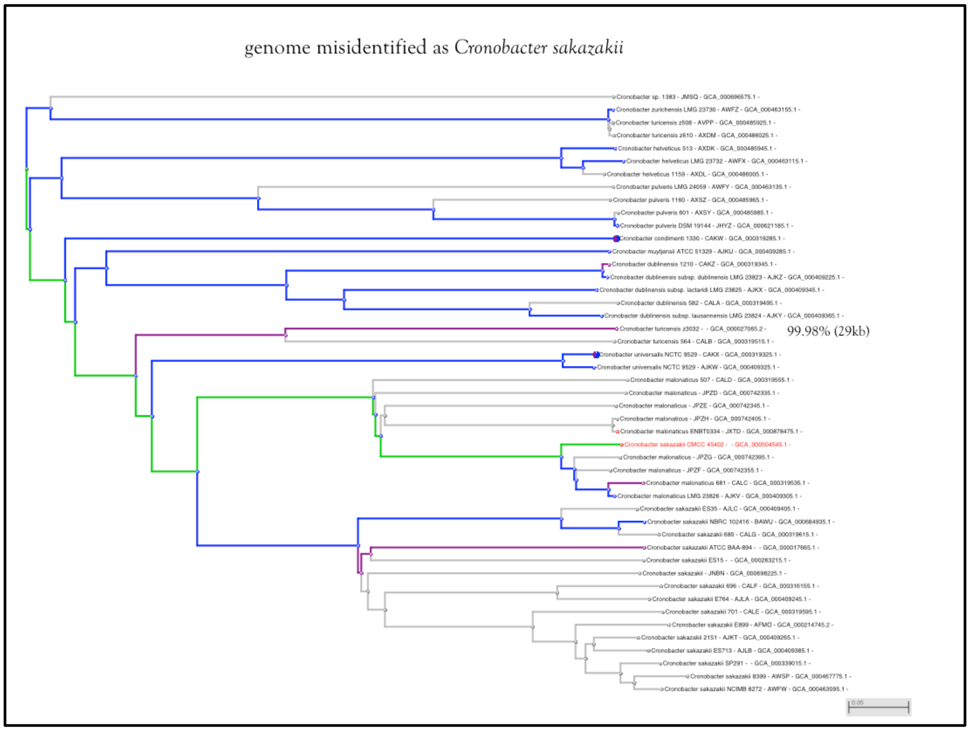

Position of the putative misidentification in the kmer tree (lineage trace in green, label in red). All trees in this presentation are neighbor-joining trees of pairwise kmer statistics, imaged in the Genome Workbench.

Lineage traces in blue lead to genomes from type (and proxytype). lineages in purple are RefSeq reference & representative genomes. We do not have a genome from type for Cronobacter turicensis, but 29kb of GenBank sequence from type identifies GCA_000027065 as the proxytype genome for this species (at 99.98% identity). There is a YouTube web tutorial on using the genome workbench tree viewer – https://youtu.be/dei0Z6HS6OM

non-genomic type sequences in GenBank

947	*Agrobacterium fabrum*
715	*Bacillus subtilis*
469	*Desulfovibrio vulgaris*
421	*Bacillus alcalophilus*
391	*Legionella pneumophila*
	...
75	*Bacillus subtilis* subsp. *spizizenii*
	...
5	560 species
4	787 species
3	1004 species
2	1648 species
1	4552 species
0	2935 species

Counts of sequence from type in GenBank (excluding components of genomes from type). We do have genomes from type for the top species on this list. The 4552 species with a single type sequence in GenBank will be almost entirely 16S sequences. Many of the 2935 species without type sequences in GenBank will be legacy names without standing in the current nomenclature.

how identical is co-identical?

4 pairs of genomes < 90% identical.
5 pairs of genomes 96% - 99% identical.
8 pairs of genomes 99% - 99.9% identical.
112 pairs of genomes 99.9% - 99.99% identical.
135 pairs of genomes 99.99% - 99.999% identical.
10 pairs of genomes > 99.999% identical.

There are 274 species for which we have more than one genome from type, often sequenced by different labs, from different culture collections. The median ANI is just over 99.99% identity, but there is a wide range of values.

81.77%	Streptomyces aureofaciens NRRL B-2657	(18.35%)	GCA_000719175
	Streptomyces aureofaciens NRRL B-2183	(20.09%)	GCA_000720845
83.13%	Pseudomonas cremoricolorata DSM 17059	(54.39%)	GCA_000425745
	Pseudomonas cremoricolorata NBRC 16634	(56.75%)	GCA_000730545
83.36%	Streptomyces sclerotialus NRRL B-2317	(36.92%)	GCA_000719105
	Streptomyces sclerotialus NRRL ISP-5269	(31.52%)	GCA_000720555
89.21%	Acinetobacter lwoffii CIP 64.10	(79.32%)	GCA_000369105
	Acinetobacter lwoffii NCTC 5866	(82.94%)	GCA_000248355
96.27%	Piscirickettsia salmonis LF-89	(79.81%)	GCA_000297215
	Piscirickettsia salmonis ATCC VR-1361	(78.96%)	GCA_000300295
96.80%	Bacillus subtilis subsp. spizizenii TU-B-10	(89.73%)	GCA_000227465
	Bacillus subtilis subsp. spizizenii ATCC 6633	(94.87%)	GCA_000177595
97.98%	Brucella inopinata BO1	(91.32%)	GCA_000662015
	Brucella inopinata BO1	(87.03%)	GCA_000182725
98.20%	Actinobacillus pleuropneumoniae str. 4074	(90.56%)	GCA_000167095
	Actinobacillus pleuropneumoniae str. 4074	(91.75%)	GCA_000178495
98.35%	Eubacterium rectale ATCC 33656	(73.93%)	GCA_000020605
	Eubacterium rectale DSM 17629	(78.28%)	GCA_000209935
99.03%	Eggerthia catenaformis OT 569	(87.13%)	GCA_000340375
	Eggerthia catenaformis DSM 20559	(87.94%)	GCA_000422605
99.45%	Edwardsiella tarda ATCC 15947	(90.67%)	GCA_000264805
	Edwardsiella tarda NBRC 105688	(92.78%)	GCA_000341505
99.47%	Mycobacterium intracellulare ATCC 13950	(95.87%)	GCA_000172115
	Mycobacterium intracellulare ATCC 13950	(97.20%)	GCA_000277125
99.87%	Cronobacter universalis NCTC 9529	(94.01%)	GCA_000319325
	Cronobacter universalis NCTC 9529	(98.19%)	GCA_000409325
99.88%	Prevotella disiens ATCC 29426	(99.60%)	GCA_000467875
	Prevotella disiens JCM 6334	(95.61%)	GCA_000613345
99.88%	Bifidobacterium angulatum DSM 20098	(99.77%)	GCA_000156635
	Bifidobacterium angulatum LMG 11039	(99.94%)	GCA_000741065
99.88%	Prochlorothrix hollandica PCC 9006	(58.29%)	GCA_000332315
	Prochlorothrix hollandica PCC 9006	(97.04%)	GCA_000341585
99.89%	Streptomyces clavuligerus ATCC 27064	(72.29%)	GCA_000148465
	Streptomyces clavuligerus ATCC 27064	(98.16%)	GCA_000154925

These are the most divergent values, including all of the cases with less that 99.9% identity. One was from inappropriate annotation in the taxonomy database – ATCC 6633 is not a type strain of Bacillus subtilis subsp. spizizenii. Others are assemblies with contamination – note that GCA_000332315 is almost twice the size of GCA_000154925 (both of which are from PCC 9006). Others are problems with strains in the culture collections – NRRL B-2657 & NRRL B-2183 both claim to be type strains of Streptomyces aureofaciens. Neither of these are actually type, and NRRL B-2657 is likely from a different species.

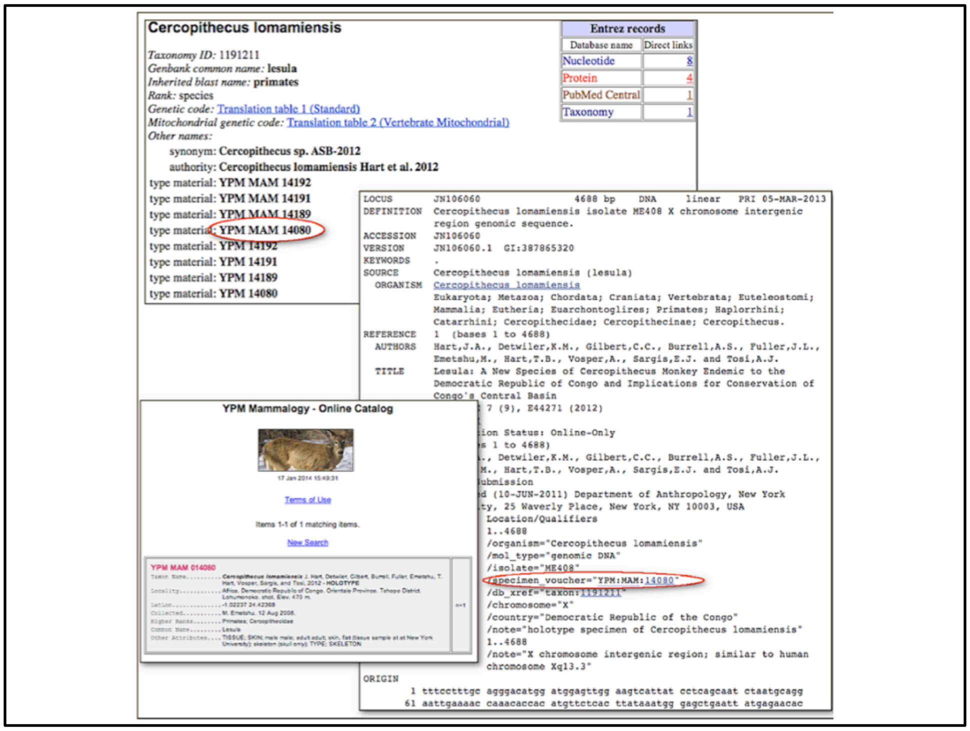

We annotate type material in the taxonomy database, and compare with annotation in the GenBank source features to flag sequences from type. Figure from (Federhen, 2015).

Types of Types

**type strain**	(P) deposited in at least two different culture collections
**neotype strain**	(P) replacement culture for a type that has been lost
**holotype**	(BZ) single name-bearing type specimen
**paratype**	(BZ) other specimens in the original type series
**neotype**	(BZ) single name-bearing specimen designated when holotype is lost
**allotype**	(Z) designated specimen of opposite sex to the holotype
**epitype**	(B) other specimen designated in subsequent type series
**isotype**	(B) duplicate specimen of the holotype
**syntype**	(BZ) one of a name-bearing series of type specimens
**isosyntype**	(B) duplicate specimen of a syntype
**lectotype**	(BZ) syntype subsequently designated as the single name-bearing type
**paralectotype**	(B) syntype specimens not designated as lectotype
**hapanotype**	(B) protest culture/collection collectively designated as holotype
**type material**	(-) type material of unknown type
**reference material**	(-) non-type material reserved for arbitrary uses
**culture from type**	(-) living culture derived from type material

Types of type annotated in the NCBI taxonomy database, taken from the bacterial (P), botanical (B) and zoological (Z) codes of nomenclature. Figure from (Federhen, 2015)

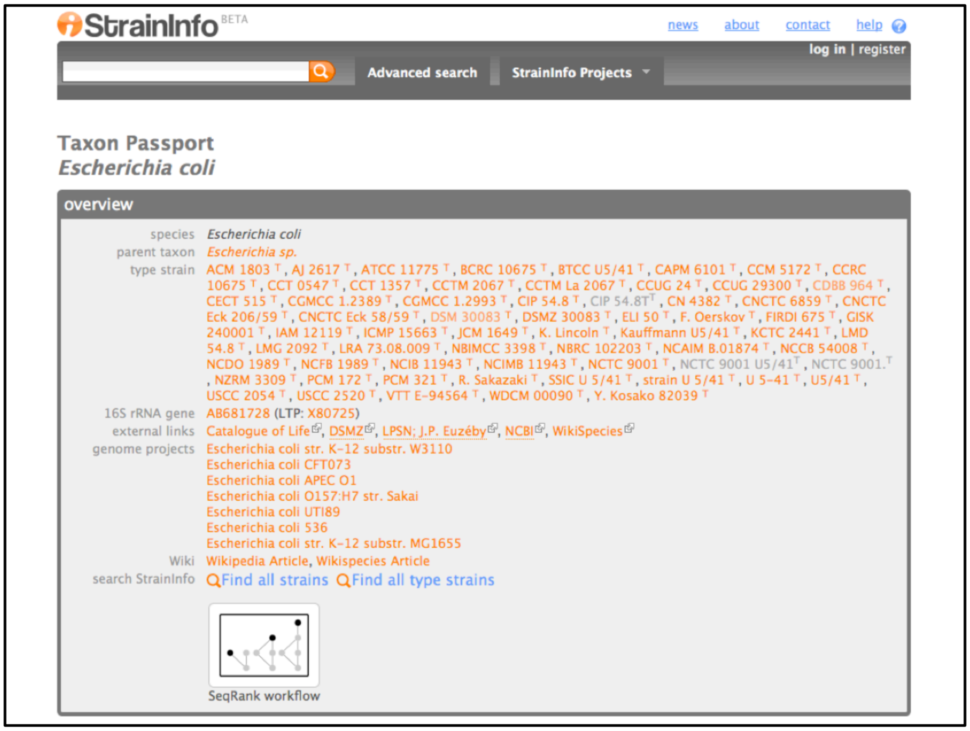

Types for Escherichia coli in StrainInfo – data derived from the corresponding culture collections.

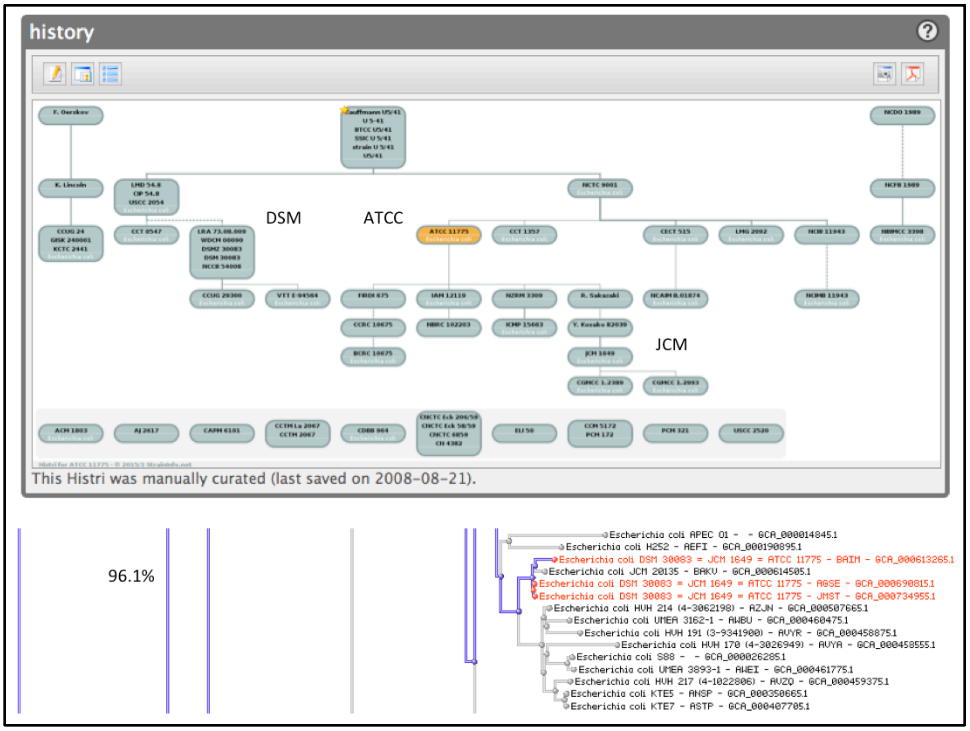

StrainInfo history diagram for the types of Escherichia coli. We have WGS genomes for three of them, from the DSM, ATCC & JCM. These are nearly identical, and form a tight cluster in the kmer tree (together with JCM 20135).

JCM 20135 is annotated as K-12 at the culture collection – either that is wrong, or our genome is not actually from JCM 20135.

Dickeya, misidentified representative genomes CP001654 & CP001655

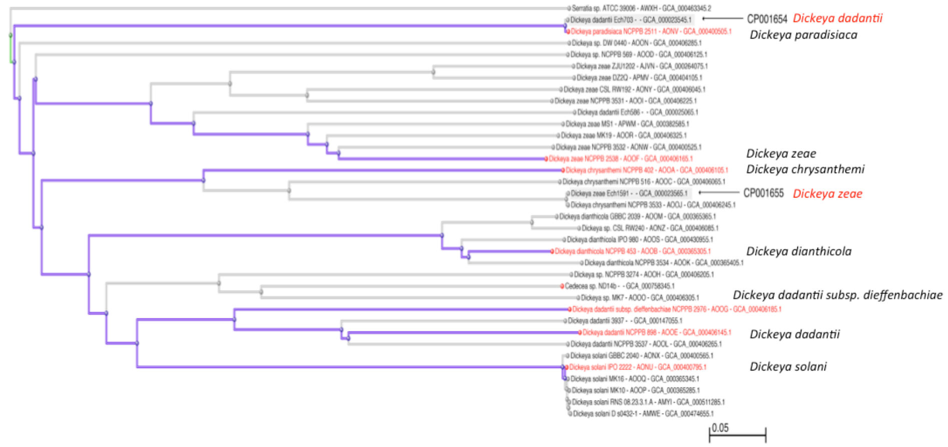

99.9945 CP001654 AONV 98.8896 99.9997 – vs. type of *Dickeya paradisiaca*
82.4630 CP001654 AOOE 51.6877 49.0712 – vs. type of *Dickeya dadantii*

99.9981 CP001655 AOOJ 98.0741 99.9407 – vs. *Dickeya chrysanthemi*, not from type
96.5888 CP001655 AOOA 82.8675 84.9573 – vs. type of *Dickeya chrysanthemi*
86.8879 |CP001655 AOOF 75.5509 79.8169 – vs. type of *Dickeya zeae*

genomes from type in the genus Dickeya, with two misidentified genomes. CP001654 was submitted as Dickeya dadantii, but is nearly identical to the type genome of Dickeya paradisiaca.
CP001655 was submitted as Dickeya zeae, but is actually Dickeya chrysanthemi. These two GenBank entries have been UNVERIFIED.

```
LOCUS       CP001654             4679450 bp    DNA     circular BCT 10-MAR-2015
DEFINITION  UNVERIFIED: Dickeya dadantii Ech703, complete genome.
ACCESSION   CP001654
VERSION     CP001654.1  GI:242129517
DBLINK      BioProject: PRJNA33069
            BioSample: SAMN02598494
KEYWORDS    UNVERIFIED.
SOURCE      Dickeya dadantii Ech703
  ORGANISM  Dickeya dadantii Ech703
            Bacteria; Proteobacteria; Gammaproteobacteria; Enterobacteriales;
            Enterobacteriaceae; Dickeya.
REFERENCE   1  (bases 1 to 4679450)
  AUTHORS   Lucas,S., Copeland,A., Lapidus,A., Glavina del Rio,T., Dalin,E.,
            Tice,H., Bruce,D., Goodwin,L., Pitluck,S., Chertkov,O., Brettin,T.,
            Detter,J.C., Han,C., Larimer,F., Land,M., Hauser,L., Kyrpides,N.,
            Mikhailova,N., Balakrishnan,V., Glasner,J. and Perna,N.T.
  CONSRTM   US DOE Joint Genome Institute
  TITLE     Complete sequence of Dickeya dadantii Ech703
  JOURNAL   Unpublished
REFERENCE   2  (bases 1 to 4679450)
  AUTHORS   Lucas,S., Copeland,A., Lapidus,A., Glavina del Rio,T., Tice,H.,
            Bruce,D., Goodwin,L., Pitluck,S., Chertkov,O., Brettin,T.,
            Detter,J.C., Han,C., Larimer,F., Land,M., Hauser,L., Kyrpides,N.,
            Mikhailova,N., Balakrishnan,V., Glasner,J. and Perna,N.T.
  CONSRTM   US DOE Joint Genome Institute
  TITLE     Direct Submission
  JOURNAL   Submitted (24-JUN-2009) US DOE Joint Genome Institute, 2800
            Mitchell Drive B310, Walnut Creek, CA 94598-1698, USA
COMMENT     GenBank staff is unable to verify source organism provided by the
            submitter.
            CP001654 was submitted as Dickeya dadantii Ech703, but it is
            99.9988% identical to the genome from Dickeya paradisiaca NCPPB
            2511 (WGS entry AONV00000000.1), the type strain of Dickeya
            paradisiaca
```

The UNVERIFIED entry for Dickeya dadantii Ech703. UNVERIFIED entries are indexed in Entrez and exchanged with the INSDC, but are not present in the default BLAST databases.

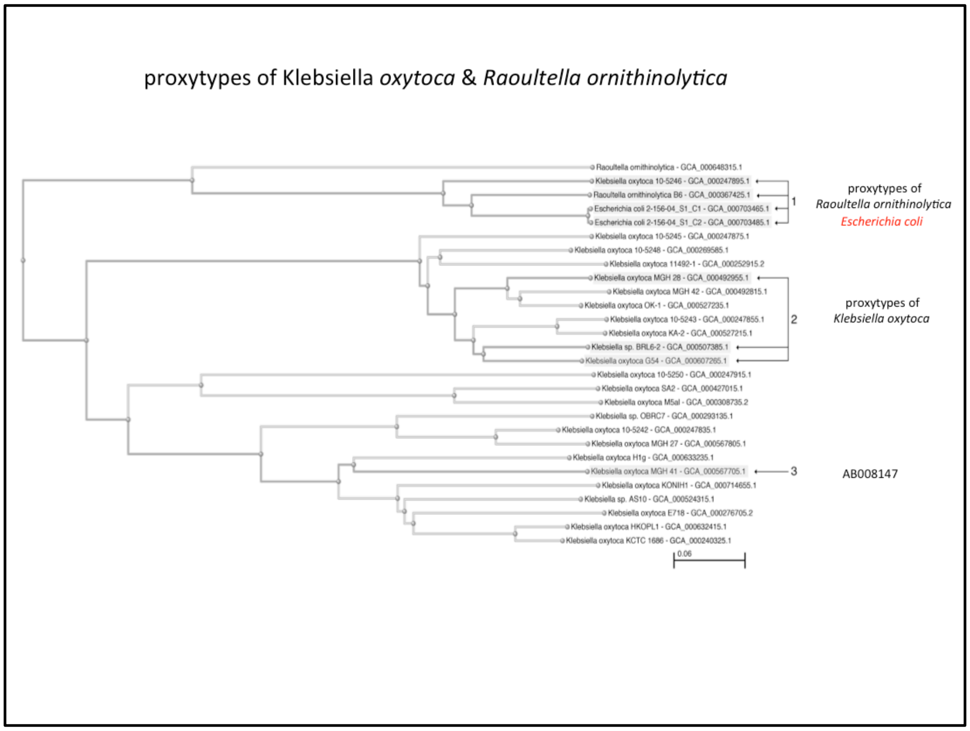

Two genomes misidentified as Escherichia coli appeared in the Raoultella/Klebsiella oxytoca clade. We don't have genomes from type for either Klebsiella oxytoca or Raoultella ornithinolytica, but we can assign proxytype genomes based on sequences from type in GenBank. AB008147 is anomalous – it claims to be type sequence from Klebsiella oxytoca, but does not behave as expected.

proxytype computation for *Raoultella ornithnolytica*

			AF129447 ATCC 31898 rpoB	AF303652 ATCC 31898 parC	JX397961 DSM 7464 tuf	AY134479 DSM 7464 tuf	AF303618 ATCC 31898 gyrA	DQ227492 DSM 7464 oriC	AY307386 ATCC 31898 blaORN1a	AY134485 DSM 7464 atpD	AB008149 JCM 6096 groES/EL
99.505	6638/6671	Raoultella ornithinolytica B6	496	319	515	789	379	289	1128	801	1922
99.505	6638/6671	Escherichia coli 2-156-04_S1_C2	494	319	515	789	380	289	1128	803	1921
99.505	6638/6671	Escherichia coli 2-156-04_S1_C1	494	319	515	789	380	289	1128	803	1921
99.49	6637/6671	Klebsiella oxytoca 10-5246	496	319	515	789	380	289	1125	801	1923
97.692	6517/6671	Raoultella ornithinolytica	487	318	510	784	380	287	1051	795	1905
94.549	4753/5027	Klebsiella oxytoca MGH 41		289	507	779	349	261		759	1809
94.49	4750/5027	Klebsiella oxytoca 10-5242		290	507	778	351	261		759	1804
94.47	4749/5027	Klebsiella oxytoca MGH 27		290	507	778	350	261		759	1804
94.45	4748/5027	Klebsiella sp. AS10		289	505	777	352	259		759	1807
94.317	5228/5543	Klebsiella oxytoca HKOPL1	473	289	507	779	352	261		759	1808
94.299	5227/5543	Klebsiella oxytoca KCTC 1686	472	289	507	779	352	261		759	1808
94.281	5226/5543	Klebsiella oxytoca KONIH1	473	289	507	779	351	261		759	1807
94.245	5224/5543	Klebsiella oxytoca E718	471	288	507	779	351	261		759	1808
94.245	5224/5543	Klebsiella oxytoca	473	289	507	779	352	258		759	1807
94.231	4737/5027	Klebsiella oxytoca M5al		291	505	775	352	257		757	1800
94.028	4912/5224	Enterobacter aerogenes KCTC 2190	471		497	764	354	258		772	1796
94.028	4912/5224	Enterobacter aerogenes EA1509E	470		497	764	354	258		775	1794
93.92	5206/5543	Klebsiella oxytoca 10-5250	478	291	506	777	349	257		755	1793
93.496	4701/5028	Klebsiella oxytoca 11492-1	472	288		768	349	260		760	1804
93.477	4700/5028	Klebsiella oxytoca 10-5245	474	288		768	349	259		761	1801
93.455	4612/4935	Enterobacter cloacae ECNIH2	474		499	756	341			763	1779
93.389	4549/4871	Enterobacter lignolyticus SCF1	466	290	504	770				755	1764
93.325	5173/5543	Klebsiella variicola At-22	469	286	497	764	340	261		762	1794
93.29	4435/4754	Klebsiella oxytoca SA2	473	291	505		352	257		757	1800
93.289	5171/5543	Klebsiella pneumoniae 342	470	286	496	763	339	262		761	1794
93.212	4600/4935	Enterobacter asburiae L1	473		497	751	341			755	1783
93.166	4867/5224	Citrobacter freundii CFNIH1	473		501	771	353	242		762	1765
93.147	4866/5224	Klebsiella pneumoniae subsp. pneumoniae MGH 7857	465		495	763	337	262		762	1782
93.126	5162/5543	Klebsiella pneumoniae subsp. pneumoniae KP5-1	470	286	494	761	340	261		762	1788
93.109	4864/5224	Klebsiella pneumoniae subsp. pneumoniae Kp13	466		495	763	334	262		762	1782
93.09	4863/5224	Klebsiella pneumoniae subsp. pneumoniae 1084	466		495	763	336	262		761	1780
93.07	4862/5224	Klebsiella pneumoniae	466		495	761	336	262		762	1780
93.051	4861/5224	Klebsiella pneumoniae subsp. pneumoniae HS11286	465		495	762	336	262		762	1779
93.051	4861/5224	Klebsiella pneumoniae CG43	465		495	763	336	261		762	1779
93.051	4861/5224	Klebsiella pneumoniae ATCC BAA-2146	465		495	762	336	262		762	1779
93.032	4860/5224	Klebsiella pneumoniae 30684/NJST258_2	465		495	762	335	262		762	1779
93.032	4860/5224	Klebsiella pneumoniae 30660/NJST258_1	465		495	762	335	262		762	1779
93.015	4887/5254	Enterobacter cloacae subsp. cloacae ATCC 13047	469	285	497	753	343			756	1784

We list nine sequences from type for Raoultella ornithinolytica (ribosomal RNAs were excluded from this computation) Counts in the table are the number of identities in the strongest BLAST HSP.

These are accumulated by target genome and sorted by %identity. The top four genomes are equally good proxytypes for Raoultella ornithinolytica (at 99.5% identity) Only one of these was actually submitted with the name Raoultella ornithinolytica.

proxytype computation for *Klebsiella oxytoca*

			EU010082 ATCC 13182 pyrG	DQ227490 DSM 5175 oriC	AF525464 ATCC 13182 albA	(AB008147) JCM 1665 groEL	AF038433 ATCC 13182 gyrA	AY367363 ATCC 13182 rpoB	EU010028 ATCC 13182 fusA	JX424964 LMG 3055 atpD	AY367380 ATCC 13182 Mdh	AF473577 ATCC 13182 bla OXY	AF052257 ATCC 13182 gyrA	JX425223 LMG 3055 infB	JX425093 LMG 3055 gyrB	JX425349 LMG 3055 rpoB	EU010055 ATCC 13182 leuS	EU010109 ATCC 13182 rpoB	U77442 ATCC 13182 rpoB	AY370849 ATCC 13182 gyrB
99.883	10206/10218	Klebsiella sp. BRL6-2	306	289	663	1872	178	1029	633	642	670	1056	589	615	742	637	642	501	512	502
99.746	10192/10218	Klebsiella oxytoca MGH 28	306	287	662	1873	178	1026	632	642	670	1056	588	615	742	636	639	500	511	502
99.746	10192/10218	Klebsiella oxytoca G54	306	289	663	1873	178	1027	633	642	670	1053	589	615	740	636	639	500	511	501
99.716	10189/10218	Klebsiella oxytoca MGH 42	306	287	666	1873	178	1026	632	642	669	1052	588	615	742	636	637	500	511	502
99.667	10184/10218	Klebsiella oxytoca OK-1	306	286	662	1873	178	1026	632	642	670	1051	588	615	742	636	637	500	511	502
99.657	10183/10218	Klebsiella oxytoca 10-5245	305	286	662	1873	177	1028	632	642	667	1053	588	613	740	637	638	500	512	503
99.648	10182/10218	Klebsiella oxytoca 11492-1	305	287	665	1872	178	1026	632	641	670	1053	588	613	740	635	636	500	510	503
99.648	10182/10218	Klebsiella oxytoca 10-5248	306	287	662	1872	178	1026	633	641	670	1054	588	613	740	635	639	499	510	501
99.609	10178/10218	Klebsiella oxytoca KA-2	305	288	661	1873	178	1026	632	642	670	1051	589	615	739	635	636	499	510	502
99.609	10178/10218	Klebsiella oxytoca 10-5243	305	288	661	1873	178	1026	632	642	670	1051	589	615	739	635	636	499	510	502
95.234	9731/10218	Klebsiella oxytoca HKOPL1	287	281	624	1915	172	1003	624	636	616	913	575	599	704	622	605	487	495	488
95.205	9728/10218	Klebsiella oxytoca KONIH1	286	281	624	1912	171	1003	623	636	617	915	574	599	702	622	606	487	495	487
95.195	9727/10218	Klebsiella oxytoca KCTC 1686	287	281	624	1915	172	1002	624	636	616	913	575	599	704	621	605	486	494	488
95.195	9727/10218	Klebsiella oxytoca	286	278	622	1912	172	1003	624	636	617	915	575	599	704	622	605	487	495	487
95.175	9725/10218	Klebsiella sp. AS10	285	279	624	1912	172	1003	624	636	616	915	575	599	701	622	605	487	495	487
95.097	9717/10218	Klebsiella oxytoca E718	285	281	623	1913	171	1001	624	636	616	912	574	599	704	620	606	485	493	487
94.981	9121/9603	Klebsiella oxytoca MGH 41	286	281	625	1927	172	1001	624	637	615	912	575		702	621	602	486	495	487
94.949	9118/9603	Klebsiella sp. OBRC7	286	281	620	1914	172	1001	624	635	614	914	575		703	620	608	487	493	485
94.949	9118/9603	Klebsiella oxytoca 10-5242	286	281	622	1914	172	1003	624	636	614	907	575		702	622	608	487	495	484
94.929	9116/9603	Klebsiella oxytoca MGH 27	286	281	622	1914	171	1003	624	636	614	907	574		702	622	608	487	495	484
94.887	9112/9603	Klebsiella oxytoca H1g	285	281	621	1924	173	1000	624	636	616	914	573		701	620	604	486	493	485
94.356	9061/9603	Klebsiella oxytoca SA2	288	279	598	1874	176	996	627	630	625	906	575		695	619	588	486	495	478
94.293	9055/9603	Klebsiella oxytoca M5al	288	279	598	1874	176	995	627	630	626	907	575		692	618	589	485	495	475
93.825	9010/9603	Klebsiella oxytoca 10-5250	289	279	592	1874	177	987	626	633	622	902	575		694	613	579	482	486	474
93.542	6142/6566	Enterobacter aerogenes UCI 48	279	262		1805	166	986	599				552		673	618	571	489	495	452
93.542	6142/6566	Enterobacter aerogenes UCI 16	279	262		1805	166	986	599				552		673	618	571	489	495	452
93.542	6142/6566	Enterobacter aerogenes UCI 15	279	262		1805	166	986	599				552		673	618	571	489	495	452
93.542	6142/6566	Enterobacter aerogenes MGH-78	279	262		1805	166	986	599				552		673	618	571	489	495	452
93.527	6141/6566	Enterobacter aerogenes UCI 46	279	262		1805	166	986	599				552		673	618	570	489	495	452
93.512	6140/6566	Enterobacter aerogenes UCI 47	279	262		1805	165	986	599				551		673	618	571	489	495	452
93.512	6140/6566	Enterobacter aerogenes MGH-77	279	262		1805	165	986	599				551		673	618	571	489	495	452
93.451	6136/6566	Enterobacter aerogenes MGH 62	279	262		1808	166	987	599				552		667	619	570	489	495	451
93.451	6136/6566	Enterobacter aerogenes MGH 61	279	260		1808	166	987	598				552		670	619	569	489	495	452
93.405	6133/6566	Enterobacter aerogenes UCI 45	279	262		1807	166	984	599				552		671	617	572	487	491	453
93.405	6133/6566	Enterobacter aerogenes UCI 28	279	262		1807	166	984	599				552		671	617	572	487	491	453
93.405	6133/6566	Enterobacter aerogenes UCI 27	279	262		1807	166	984	599				552		671	617	572	487	491	453
93.36	6130/6566	Enterobacter aerogenes FGI35	277	261		1809	166	985	598				552		670	618	570	487	494	452
93.315	6533/7001	Citrobacter koseri ATCC BAA-895	272	229		1756		993	617	611			537	570	662	616		487	492	447
93.276	7297/7823	Enterobacter aerogenes KCTC 219(	279	262		1807	166	986	599	608			551	549	671	619	572	487	495	453
93.212	7292/7823	Enterobacter aerogenes EA1509E	279	262		1805	165	985	599	606			551	549	673	617	571	489	494	452
92.91	5766/6206	Enterobacter cloacae P101	264			1776		987	588	610			528	554	650	613		484	488	

We list more sequences from type for Klebsiella oxytoca, and in this case there is a single best candidate for proxytype – the WGS genome ARVT, submitted as Klebsiella sp. BRL6-2. Note that AB008147(fourth column, in parenthesis) behaves differently from the rest of these sequences. The strongest hits for this sequence appear in the middle of the table, peaking at Klebsiella oxytoca MGH 41.

proxytype computation for *Enterococcus faecium*

100	-	-	AF124222	(X87180)	X87181	(AF029771)	(AF029770)	AB075741	AF515643	HQ611243	AJ843271	EU021375	GU457278	AF375581	AF417582	AJ295305	AJ843428	AJ843486
			ATCC 19434	ATCC 19434	ATCC 19434	ATCC 19434	ATCC 19434	JCM 5804	DSM 20477	ATCC 19434	LMG 11423	CIP 103014	ATCC 19434	LMG 11423	ATCC 19434	LMG 11423	LMG 11423	LMG 11423
			tuf	ITS	ITS	IS1485	IS1485	enterocin	rpoB	rpoB	atpA	groEL	atpD	tmRNA	groES/EL	23S	pheS	rpoA
			751 bp.	344 bp.	94 bp.	758 bp.	195 bp.	197 bp.	323 bp.	1177 bp.	1102 bp.	757 bp.	718 bp.	318 bp.	1904 bp.	2914 bp.	455 bp.	700 bp.
99.901	15082/15097	Enterococcus faecium LCT-EF128	746	218	91	713	195	196		1177	1102	757	718	318	1904		455	700
99.9	17993/18011	Enterococcus faecium EnGen0007	746	342	91	753	195	196		1177	1102	757	718	318	1904	2910	455	700
99.876	14516/14534	Enterococcus sp. GMD4E	746	152			39			1177		757		318	1903	2909	455	700
99.848	13119/13139	Enterococcus faecium UC10237	746	220		714	157			1177	1094	757	718	318	1904		455	700
99.843	17786/17814	Enterococcus faecium EnGen0175	746	343	91	753	195			1177	1091	757	718	318	1904	2910	455	700
99.842	14576/14599	Enterococcus durans FB129-CNAB-4	746	343	91	753	195			1177	1093	757	718	318	1904		455	700
99.84	17485/17513	Enterococcus faecium EnGen0031	746	343	91	753	195			1177	1091	757	718	318	1904	2910	455	700
99.837	17785/17814	Enterococcus faecium EnGen0149	746	340	91	753	195			1177	1091	757	718	318	1904	2909	455	700
99.83	16420/16448	Enterococcus faecium LA4B-2	746	269	91					1177	1094	757	718	318	1904	2909	455	700
99.828	17980/18011	Enterococcus faecium ATCC 8459	746	342	91	753	195	193		1177	1094	757	718	318	1903	2910	455	700
99.826	17783/17814	Enterococcus faecium EnGen0018	746	342	91	753	42			1176	1090	757	718	318	1904	2910	455	700
99.82	17782/17814	Enterococcus faecium E1071	746	270	91		157			1177	1094	757	718	318	1904	2909	455	700
99.819	17678/17710	Enterococcus faecium NRRL B-2354	746	343	91	753	195	193		1177	1094	757	718	318	1904	2909	455	700
99.819	11599/11620	Enterococcus sp. GMD3E	746				39			1177		757		318	1903		455	700
99.814	17687/17720	Enterococcus faecium EnGen0178	746	341			51			1177	1093	756	717	318	1903	2909	455	699
99.804	17779/17814	Enterococcus faecium NEF1	746	341	91		51			1177	1093	756	717	318	1903	2910	455	699
99.8	17478/17513	Enterococcus faecium EnGen0016	746	341	91		40			1177	1093	756	717	318	1903	2910	454	699
99.799	13403/13430	Enterococcus faecium CRL1879	746	266	91	753	195	192		1177	1094	757	718	318	1904		455	700
99.798	17778/17814	Enterococcus faecium EnGen0030	746	342	91		40			1177	1093	756	717	318	1903	2910	455	699
99.793	16414/16448	Enterococcus faecium EnGen0254	746		91		40			1177	1093	756	717	318	1903	2910	455	699
99.792	17777/17814	Enterococcus faecium EnGen0377	746	342	91		40			1177	1089	757	718	318	1904	2910	455	699
99.792	17777/17814	Enterococcus faecium EnGen0376	746	342	91		40			1177	1089	757	718	318	1904	2910	455	699
99.792	17777/17814	Enterococcus faecium EnGen0321	746	342	91		40			1177	1089	757	718	318	1904	2910	455	699
99.792	17777/17814	Enterococcus faecium EnGen0180	746	342	91		40			1177	1089	757	718	318	1903	2910	455	699
99.789	17476/17513	Enterococcus faecium DO	746	342	91		41			1177	1093	756	717	318	1903	2910	454	699
99.787	17776/17814	Enterococcus faecium EnGen0375	746	342	91		40			1177	1089	757	718	318	1904	2909	455	699

One more illustrative proxytype calculation. In this case AF515643 doesn't make the cut with any of the genomes in the table. HQ611243 is also from rpoB, and behaves as expected. his turned out to be a strain mix-up in the submitter's lab, and has been suppressed.

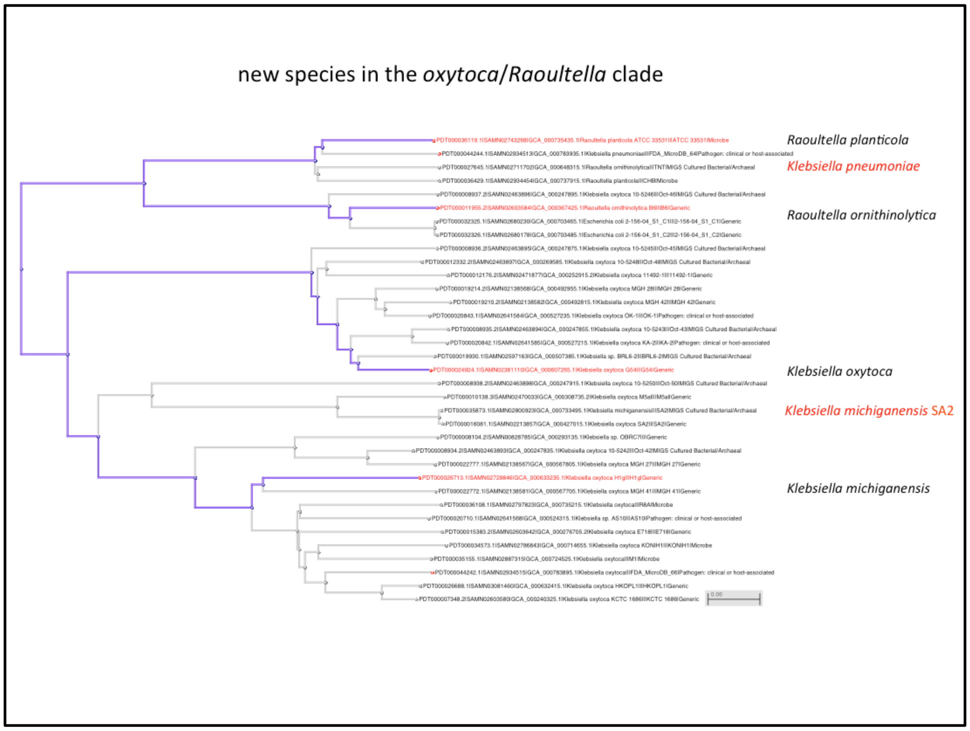

After the publication of (Federhen 2015) we looked at same region again. Several changes had occurred.

A type genome for Raoultella planticola appeared, along with another misidentified genome (Klebsiella pneumoniae this time).

In addition, someone resequenced strain SA2 at higher coverage, and submitted it as Klebsiella michiganensis instead of Klebsiella oxytoca.

proxytype calculation for *Klebsiella michiganensis*

			JQ269337 strain W14 834 bp rpoB	JQ070300 strain W14 1395 bp 16S	JQ990329 strain W14 625 bp. gyrA	JQ284304 strain W14 978 bp gyrB
99.347	3804/3829	Klebsiella oxytoca H1g	831 834 0	1382 1392 0	626 626 0	965 977 1
99.19	3798/3829	Klebsiella oxytoca MGH 41	830 834 0	1382 1392 0	622 626 0	964 977 1
99.138	2416/2437	Klebsiella sp. OBRC7	830 834 0		622 626 0	964 977 1
99.112	3795/3829	Klebsiella oxytoca KCTC 1686	828 834 0	1382 1392 0	621 626 0	964 977 1
99.086	3794/3829	Klebsiella oxytoca KONIH1	829 834 0	1382 1392 0	620 626 0	963 977 1
99.06	3793/3829	Klebsiella sp. AS10	829 834 0	1382 1392 0	621 626 0	961 977 1
99.06	3793/3829	Klebsiella oxytoca MGH 27	829 834 0	1380 1392 0	621 626 0	963 977 1
99.034	3792/3829	Klebsiella oxytoca HKOPL1	829 834 0	1378 1392 0	621 626 0	964 977 1
99.034	3792/3829	Klebsiella oxytoca 10-5242	829 834 0	1378 1392 0	622 626 0	963 977 1
99.034	3792/3829	Klebsiella oxytoca	829 834 0	1380 1392 0	621 626 0	962 977 1
98.903	3787/3829	Klebsiella oxytoca E718	827 834 0	1378 1392 0	620 626 0	962 977 1
97.806	3745/3829	Klebsiella oxytoca SA2	809 834 0	1382 1392 0	609 626 0	945 977 1
97.806	3745/3829	Klebsiella michiganensis	809 834 0	1382 1392 0	609 626 0	945 977 1
97.728	3742/3829	Klebsiella oxytoca M5al	809 834 0	1382 1392 0	609 626 0	942 977 1
97.257	3723/3828	Klebsiella oxytoca 10-5250	801 833 0	1379 1392 0	607 626 0	936 977 1
96.911	3702/3820	Klebsiella sp. BRL6-2	805 834 0	1375 1393 2	610 626 0	912 967 0
96.859	3700/3820	Klebsiella oxytoca MGH 42	805 834 0	1374 1393 2	609 626 0	912 967 0
96.859	3700/3820	Klebsiella oxytoca MGH 28	805 834 0	1374 1393 2	609 626 0	912 967 0
96.859	3700/3820	Klebsiella oxytoca G54	804 834 0	1375 1393 2	610 626 0	911 967 0
96.859	3700/3820	Klebsiella oxytoca 10-5248	805 834 0	1374 1393 2	610 626 0	911 967 0
96.859	3700/3820	Klebsiella oxytoca 10-5243	805 834 0	1373 1393 2	610 626 0	912 967 0
96.754	3696/3820	Klebsiella oxytoca 10-5245	804 834 0	1368 1393 2	611 626 0	913 967 0
96.702	3694/3820	Klebsiella oxytoca OK-1	805 834 0	1368 1393 2	609 626 0	912 967 0
96.702	3694/3820	Klebsiella oxytoca KA-2	805 834 0	1367 1393 2	610 626 0	912 967 0
96.181	2745/2854	Enterococcus faecalis SDVK1A	799 834 0	1365 1394 2	581 626 0	
96.181	2745/2854	Citrobacter freundii UCI 32	798 834 0	1366 1394 2	581 626 0	

The proxytype calculation for Klebsiella michiganensis, based on four sequences published with the original description.

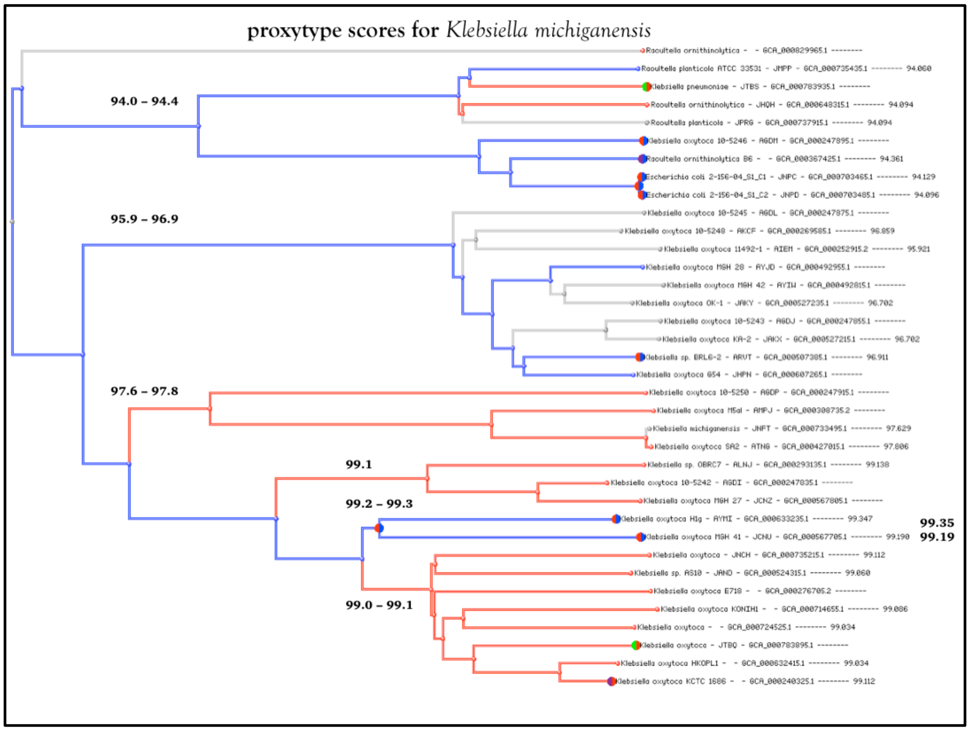

The proxytype scores for Klebsiella michiganensis are displayed on the kmer tree. Peak scores cluster around Klebsiella oxytoca H1g, and they decay in a manner consistent with the nested clades of the kmer tree.

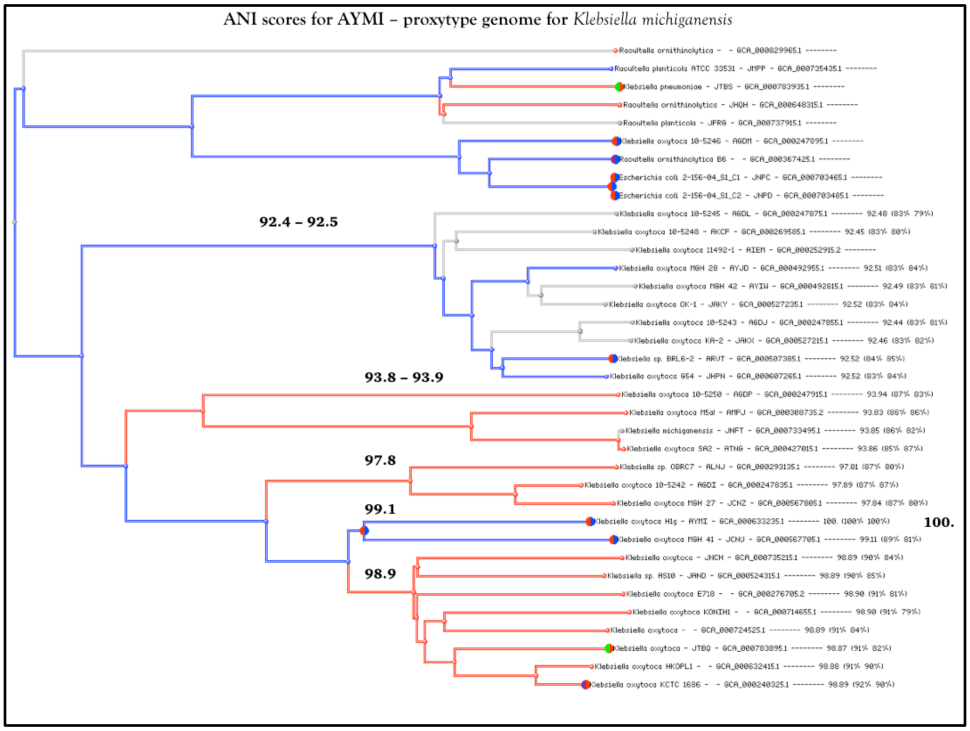

ANI neighboring scores (greater than 92%) with respect to the genome AYMI from Klebsiella oxytoca H1g, the putative proxytype genome for Klebsiella michiganensis. Traces in blue represent genomes from type & proxytype, traces in red represent genomes which would get new taxonomic identifications in this analysis. Note that Klebsiella oxytoca was a broad species (spanning 92% ANI) and that Klebsiella michiganensis as envisioned here is still a broad species (spanning 93.5% ANI).

ANI_cutoff values in Taxonomy

Acetobacter pasteurianus	92.5
Acinetobacter pittii	92.5
Aeromonas allosaccharophila	95.0
Aeromonas veronii	94.0
Bacillus subtilis	93.3
Delftia acidovorans	98.5
Delftia tsuruhatensis	98.5
Helicobacter pylori	91.0
Klebsiella michiganensis	93.5
Listeria monocytogenes	92.4
Mycobacterium africanum	99.9
Mycobacterium bovis	99.9
Mycobacterium tuberculosis	99.9
Prochlorococcus marinus	78.0
Raoultella ornithinolytica	96.5
Raoultella planticola	96.5
Rhodococcus fascians	80.0

Some current species concepts span much more (or much less) than the default rule-of-thumb 96% ANI. We can set this value on a species-by-species basis in the taxonomy database.

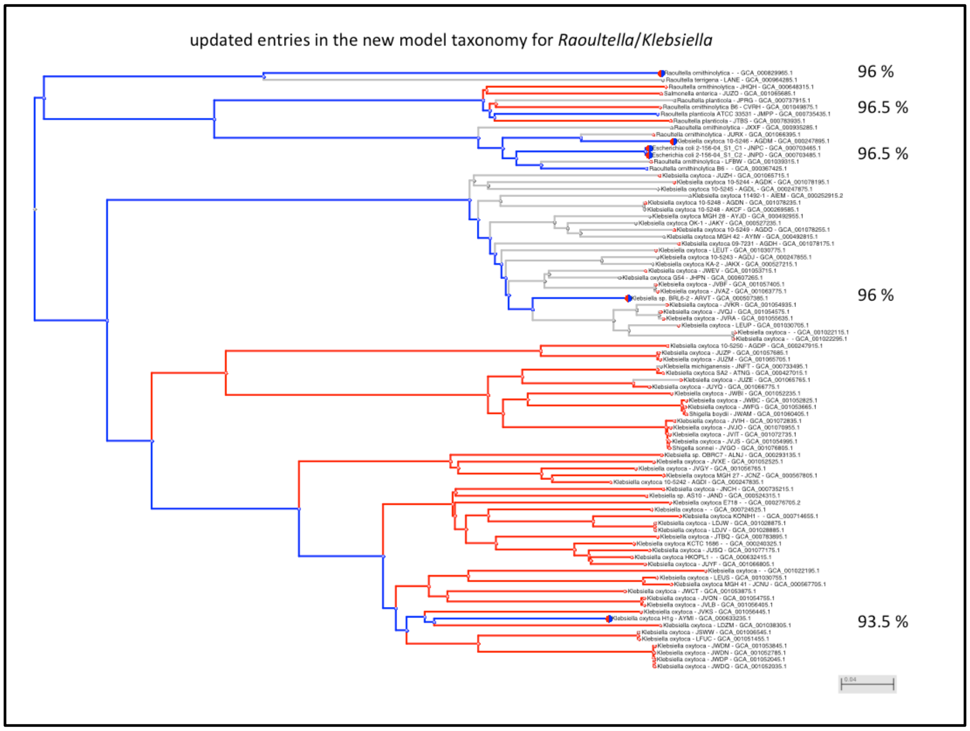

There are five species here now, one with type and four with proxytype genomes. The default ANI cutoff values work for terrigena & oxytoca. The types of Raoultella ornithinolytica & planticola are a little bit closer than default, so those ANI cutoffs have to be set a little bit higher. And the ANI cutoff for michiganensis has to be set lower, in order to effectively split the original species oxytoca in two (and to pick up our only current genome submitted as michiganensis).

(2)	*Escherichia coli*	→ *Raoultella ornithinolytica*
(1)	*Klebsiella pneumoniae*	→ *Raoultella planticola*
(1)	*Salmonella enterica*	→ *Raoultella planticola*
(1)	*Shigella boydii*	→ *Klebsiella michiganensis*
(1)	*Shigella sonnei*	→ *Klebsiella michiganensis*
(2)	*Raoultella ornithinolytica*	→ *Raoultella planticola*
(1)	*Raoultella ornithinolytica*	→ *Raoultella terrigena*
(1)	*Klebsiella oxytoca*	→ *Raoultella ornithinolytica*
(1)	*Klebsiella* sp.	→ *Klebsiella oxytoca*
(43)	*Klebsiella oxytoca*	→ *Klebsiella michiganensis*
(2)	*Klebsiella* sp.	→ *Klebsiella michiganensis*

Counts of genomes with taxonomic updates in the new model. The first set is egregious misidentifications, where the submitted identification falls outside of the Raoultella/Klebsiella clade. The second set is misidentifications within the clade. And the final set contains updates necessitated by the description of a new species (Klebsiella michiganensis Saha et al. 2013, pmid 23053492).

```
LOCUS       JHQH01000000              39 rc    DNA     linear   BCT 18-JUN-2014
DEFINITION  Raoultella planticola, whole genome shotgun sequencing project.
ACCESSION   JHQH00000000

SOURCE      Raoultella planticola (previously Raoultella ornithinolytica)

COMMENT     ##Taxonomic-Update-Data-START##
            Query            :: GCA_000648315.1
            New Taxon        :: Raoultella planticola
            Subject          :: GCA_000734435.1
            Status           :: type
            Identity         :: 99.440%
            Query Coverage   :: 90%
            Subject Coverage :: 90%

            Query            :: GCA_000648315.1
            Old Taxon        :: Raoultella ornithinolytica
            Subject          :: GCA_000367425.1
            Status           :: proxytype
            Identity         :: 96.234%
            Query Coverage   :: 86%
            Subject Coverage :: 91%
```

This genome was submitted as Raoultella ornithinolytica, but should actually be identified as Raoultella planticola. The structured comment presents the ANI neighboring data that supports this taxonomic correction.

```
LOCUS       JNPC01000000              57 rc    DNA     linear   BCT 11-JUN-2014
DEFINITION  Raoultella ornithinolytica, whole genome shotgun sequencing
            project.
ACCESSION   JNPC00000000

SOURCE      Raoultella ornithinolytica (previously Escherichia coli)

COMMENT     ##Proxytype-Designation-Data-START##
            Taxon           :: Raoultella ornithinolytica
            Assembly        :: GCA_000703465.1
            Accessions      :: AF129447 AF303652 JX397961 AY134479 AF303618
                               DQ227492 AY307386 AY134485 A008149
            Identity        :: 99.505%
            Counts          :: 6638/6671

            ##Taxonomic-Update-Data-START##
            Query            :: GCA_000703465.1
            New Taxon        :: Raoultella ornithinolytica
            Subject          :: GCA_000703465.1
            Status           :: proxytype
            Identity         :: 100.000%
            Query Coverage   :: 100%
            Subject Coverage :: 100%

            Query            :: GCA_000703465.1
            Old Taxon        :: Escherichia coli
            Subject          :: GCA_000690815.1
            Status           :: type
            Identity         :: 82.327%
            Query Coverage   :: 38%
            Subject Coverage :: 43%
```

In this case, the misidentified genome itself is one of the co-proxytype genomes of the destination species name (Raoultella ornithinolytica). We present the supporting data for the proxytype designation in a separate structured comment. After that, the first block of the taxonomic update comment is a self-comparison.

```
LOCUS       AYMI01000000             68 rc    DNA     linear   BCT 18-APR-2014
DEFINITION  Klebsiella michiganensis H1g, whole genome shotgun sequencing project.
ACCESSION   AYMI00000000

SOURCE      Klebsiella michiganensis (previously Klebsiella oxytoca)

COMMENT     ##Taxonomic-Publication-Data-START##
            Name           :: Klebsiella michiganensis
            Authority      :: Saha et al. 2013
            Effective Date :: 06-OCT-2012
            Valid Date     :: 01-MAR-2013
            Type           :: strain 14; DSM 2544; ATCC BAA-2403
            Accessions     :: JQ070300 JQ269337 JQ284304 JQ990329
            Authors        :: Saha,R., Farrance,C.E., Verghese,B.,
                              Hong,S. and Donofrio,R.S.
            Title          :: Klebsiella michiganensis sp. nov., A New
                              Bacterium Isolated from a Tooth Brush Holder
            Journal        :: Curr. Microbiol. 66 (1), 72-78 (2013)
            pmid           :: 23053492

            ##Proxytype-Designation-Data-START##
            Taxon           :: Klebsiella michiganensis
            Assembly        :: GCA_000633235.1
            Accessions      :: JQ070300 JQ269337 JQ284304 JQ990329
            Identity        :: 99.347%
            Counts          :: 3804/3829

            ##Taxonomic-Update-Data-START##
            Query            :: GCA_000633235.1
            New Taxon        :: Klebsiella michiganensis
            Subject          :: GCA_000633235.1
            Status           :: proxytype
            Identity         :: 100.000%
            Query Coverage   :: 100%
            Subject Coverage :: 100%

            Query            :: GCA_000633235.1
            Old Taxon        :: Klebsiella oxytoca
            Subject          :: GCA_000507385.1
            Status           :: proxytype
            Identity         :: 92.764%
            Query Coverage   :: 84%
            Subject Coverage :: 85%
```

Here is the supporting information for the designation of a proxytype genome for a species that is newly described in the literature. It would be useful to display some structured data about the new species.

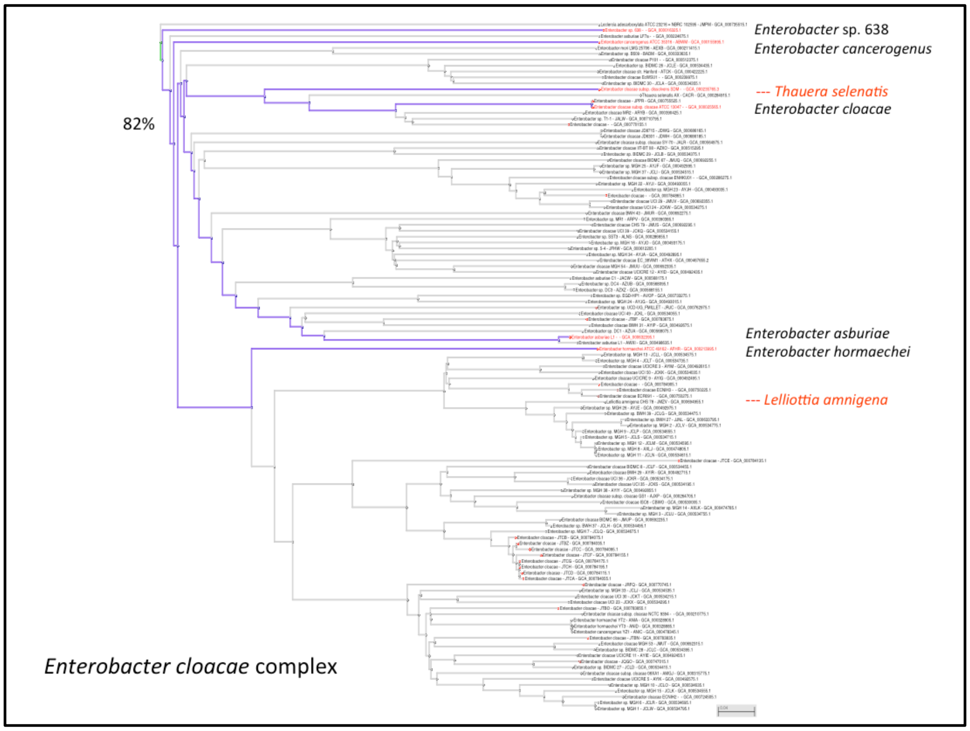

The Enterobacter cloacae complex spans 82% ANI, a very broad range. These are medically significant bacteria, and clinicians are trained to call everything here Enterobacter cloacae. A few genomes from type were submitted with different names (Enterobacter asburiae, Enterobacter hormaechei) and Enterobacter cancerogenus just outside of the cloacae complex. There are two interlopers. The WGS genome CACR (submitted as Thauera selenatis) was massively contaminated with sequence from Enterobacter cloacae, and has been suppressed. The WGS genome JMZV (submitted as Lelliottia amnigena) appears to be a simple misidentification.

proxytype table for *Lelliottia amnigena*

			JX424870<br>LMG 2784<br>642 bp.<br>atpD	JX425259<br>LMG 2784<br>637 bp.<br>rpoB	EU569334<br>DSM 4486<br>1320 bp.<br>thdF	EU569427<br>DSM 4486<br>1353 bp.<br>recN	AB682273<br>NBRC 105700<br>1402 bp.<br>16S	AB008140<br>JCM 1237<br>1930 bp.<br>GroES	JX425129<br>LMG 2784<br>615 bp.<br>infB	EU569537<br>DSM 4486<br>926 bp.<br>rpoA	AJ487974<br>CIP 103169<br>1739 bp.<br>ampC	AB004749<br>JCM 1237<br>1449 bp.<br>16S	JX425000<br>LMG 2784<br>742 bp.<br>gyrB
97.022	12347/12726	Enterobacter sp. 638	628 641 0	625 637 0	1241 1320 0	1281 1350 0	1401 1402 0	1877 1930 0	598 615 0	913 926 0	1612 1714 1	1445 1449 1	726 742 0
94.231	7841/8321	Enterobacter cloacae P101	608 641 0	586 637 0			1381 1402 0	1747 1930 17	560 615 0	895 923 0		1426 1449 1	638 724 0
94.198	7841/8324	Enterobacter cloacae EcWSU1	606 641 0	586 637 0			1381 1402 0	1747 1930 17	560 615 0	898 926 0		1426 1449 1	637 724 0
94.128	7806/8293	Enterobacter aerogenes EA1509E	602 641 0	584 637 0			1387 1402 0	1738 1914 10	546 612 0	887 926 0		1432 1449 1	630 712 0
93.767	7898/8423	Enterobacter asburiae C1	602 638 0	587 637 0	1137 1309 0		1378 1402 0	1761 1929 16		896 926 0	116 133 1	1421 1449 1	
93.7	7779/8302	Enterobacter sp. MGH 16	599 638 0	586 637 0	1137 1320 0		1381 1402 0	1756 1930 18		897 926 0		1423 1449 1	
93.699	7212/7697	Klebsiella pneumoniae subsp. pneumoniae	600 642 0	578 637 0			1374 1402 0	1732 1931 11		888 926 0		1420 1449 1	620 710 0
93.686	7211/7697	Klebsiella pneumoniae subsp. pneumoniae MGH 78578	600 642 0	579 637 0			1374 1402 0	1733 1931 11		888 926 0		1420 1449 1	617 710 0
93.658	7295/7789	Enterobacter cloacae CHS 79		589 637 0	1134 1320 0		1378 1396 0	1760 1930 18		897 926 0	113 131 2	1424 1449 1	
93.658	7192/7679	Klebsiella pneumoniae subsp. pneumoniae KPNIH27	600 642 0	577 637 0			1373 1402 0	1716 1913 8		888 926 0		1419 1449 1	619 710 0
93.653	7894/8429	Enterobacter cloacae UCICRE 12	599 638 0	586 637 0	1135 1320 0		1380 1396 0	1762 1930 18		896 926 0	113 131 2	1423 1451 3	
93.647	7208/7697	Klebsiella pneumoniae subsp. pneumoniae KPR0928	600 642 0	577 637 0			1374 1402 0	1732 1931 11		888 926 0		1420 1449 1	617 710 0
93.647	7208/7697	Klebsiella pneumoniae subsp. pneumoniae KPNIH10	600 642 0	577 637 0			1374 1402 0	1732 1931 11		888 926 0		1420 1449 1	617 710 0
93.647	7208/7697	Klebsiella pneumoniae subsp. pneumoniae KPNIH1	600 642 0	577 637 0			1374 1402 0	1732 1931 11		888 926 0		1420 1449 1	617 710 0
93.647	7208/7697	Klebsiella pneumoniae 30684/NJST258_2	600 642 0	577 637 0			1374 1402 0	1732 1931 11		888 926 0		1420 1449 1	617 710 0
93.634	7207/7697	Klebsiella pneumoniae subsp. pneumoniae Kp13	600 642 0	579 637 0			1373 1402 0	1731 1931 11		888 926 0		1419 1449 1	617 710 0
93.634	7207/7697	Klebsiella pneumoniae subsp. pneumoniae KPNIH24	600 642 0	577 637 0			1374 1402 0	1732 1931 11		888 926 0		1420 1449 1	616 710 0
93.632	7190/7679	Klebsiella pneumoniae subsp. pneumoniae NTUH-K204	599 642 0	578 637 0			1373 1402 0	1715 1913 8		888 926 0		1419 1449 1	618 710 0
93.621	7206/7697	Klebsiella pneumoniae JM45	600 642 0	577 637 0			1373 1402 0	1732 1931 11		888 926 0		1419 1449 1	617 710 0
93.593	7187/7679	Klebsiella pneumoniae CG43	598 642 0	577 637 0			1373 1402 0	1714 1913 8		888 926 0		1419 1449 1	618 710 0
93.543	7200/7697	Klebsiella pneumoniae 30660/NJST258_1	600 642 0	577 637 0			1370 1402 0	1732 1931 11		888 926 0		1416 1449 1	617 710 0
93.541	8545/9135	Enterobacter sp. DC3	604 638 0	588 637 0	1139 1309 0		1382 1402 0	1760 1929 16		896 926 0	116 133 1	1428 1449 1	632 712 0
93.528	7182/7679	Klebsiella pneumoniae subsp. pneumoniae 1084	599 642 0	578 637 0			1369 1402 1	1715 1913 8		888 926 0		1415 1449 2	618 710 0
93.519	8543/9135	Enterobacter sp. DC4	604 638 0	588 637 0	1139 1309 0		1381 1402 0	1760 1929 16		896 926 0	116 133 1	1427 1449 1	632 712 0
93.517	7198/7697	Klebsiella pneumoniae subsp. pneumoniae PittNDM01	600 642 0	576 637 0			1370 1402 0	1730 1931 11		888 926 0		1416 1449 1	618 710 0
93.517	7198/7697	Klebsiella pneumoniae subsp. pneumoniae HS11286	600 642 0	577 637 0			1369 1402 1	1732 1931 11		888 926 0		1415 1449 2	617 710 0
93.517	7198/7697	Klebsiella pneumoniae ATCC BAA-2146	600 642 0	577 637 0			1369 1402 0	1732 1931 11		888 926 0		1415 1449 1	617 710 0
93.492	8462/9051	Enterobacter sp. MGH 23	598 638 0	587 637 0	1136 1321 2		1384 1402 0	1757 1930 18	557 615 0	898 926 0	116 133 1	1429 1449 1	
93.464	7865/8415	Enterobacter cloacae BIDMC 67	599 638 0		1126 1321 2		1383 1402 0	1757 1930 18	557 615 0	898 926 0	117 134 2	1428 1449 1	

The proxytype analysis for Lelliottia amnigena identifies CP000653 (submitted as "Enterobacter sp. 638") as the proxytype genome for Lelliottia amnigena. This makes a strong prediction, that our only genome currently identified as Lelliottia amnigena is misidentified, and that the type genome (when we get one) will fall somewhere on the long branch leading to Enterobacter sp. 638 (just outside of the Enterobacter cloacae complex). Note that 97.0% identity is not a very strong score for a proxtype calculation (the queries include 16S and protein-coding regions) but it is much stronger than the next best hits. Proxytype scores run higher than genome-wide ANI scores.

Lelliottia amnigena **NBRC 105700** ANI neighboring table

ANIb

	L. amnigena NBRC 105700	Enterobacter sp. 638	E. hormaechei ATCC 49162	L. amnigena CHS 78	E. asburiae L1	E. cloacae ATCC 13047	E. cloacae EcWSU1	E. cancerogenus ATCC 35316	E. aerogenes EA1509E	K. pneumoniae MGH 78578
L. amnigena NBRC 105700	---	93.64	82.25	82.26	82.45	82.19	82.11	82.25	77.67	77.68
Enterobacter sp. 638	93.65	---	82.44	82.53	82.57	82.27	82.29	82.37	77.83	77.89
E. hormaechei ATCC 49162	82.22	82.20	---	94.12	87.40	86.66	86.16	86.13	78.56	79.27
L. amnigena CHS 78	82.35	82.50	94.24	---	87.47	86.90	86.29	86.19	78.93	79.55
E. asburiae L1	82.50	82.55	87.45	87.46	---	88.41	88.26	86.42	78.93	79.30
E. cloacae ATCC 13047	82.07	82.09	86.60	86.72	88.26	---	87.47	85.58	78.45	79.60
E. cloacae EcWSU1	82.17	82.19	86.21	86.20	88.20	87.58	---	85.75	78.42	78.73
E. cancerogenus ATCC 35316	82.22	82.27	86.18	86.18	86.41	85.76	85.79	---	78.68	79.17
E. aerogenes EA1509E	77.85	77.80	78.66	78.81	78.81	78.46	78.35	78.64	---	84.51
K. pneumoniae MGH 78578	77.73	77.67	79.20	79.39	79.12	79.23	78.58	78.97	84.33	---

unpublished draft genome from Fujita Nobuyuki, ANIb from JSpecies

Fortuitously, a bioproject was registered in 2012 for the type genome of Lelliottia amnigena. This genome has not been finished, but the submitter agreed to share the ANI neighboring statistics of their current assembly.

The type genome of Lelliottia amnigena is indeed closest to Enterobacter sp. 638 (at 93.64% ANI). Note that our current genome submitted as Lelliottia amnigena is closest to the type of Enterobacter hormaechei (at 94.2% ANI).

This provides strong support for the proxtype analysis outlined above.

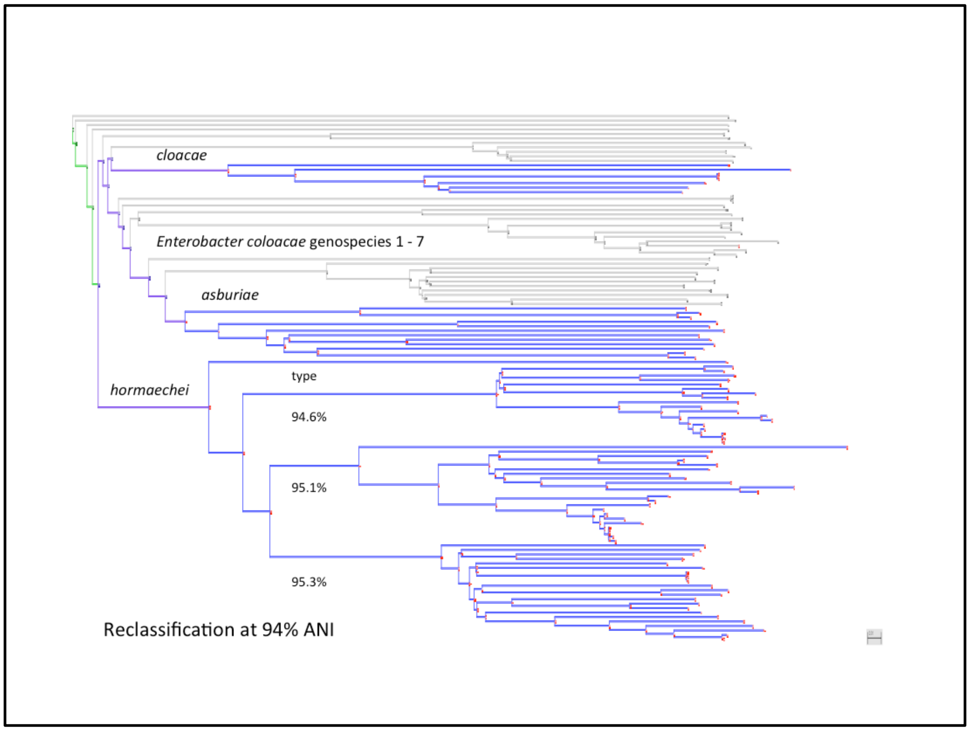

Enterobacter cloacae complex partitioned at 94% ANI with respect to the existing genomes from type. Enterobacter cloacae sensu stricto is represented by a very small clade. Several clades of genomes in the cloacae complex are less than 94% ANI from the three type genomes, and could be given informal clade names.

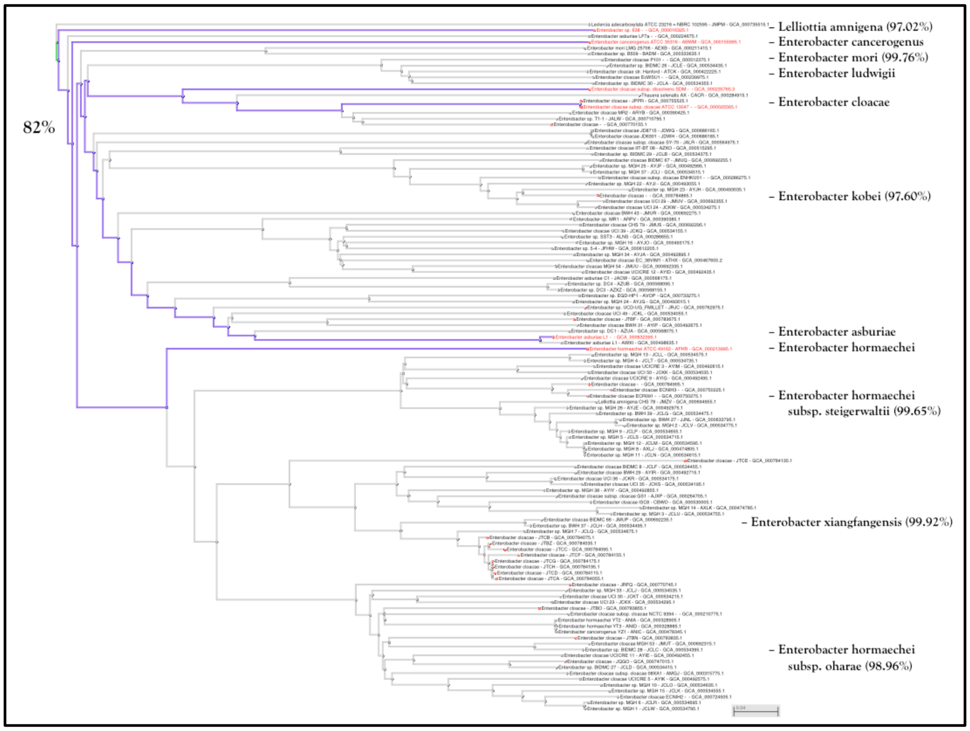

If we compute proxytypes for all of the species of Enterobacter without genomes from type, formal names can be assigned to several more clades. These include Enterobacter mori, Enterobacter kobei, Enterobacter xiangfangensis, and two subspecies of Enterobacter hormaechei that are effectively but not validly published. Note that the species Enterobacter hormaechei is paraphyletic in this labeling.

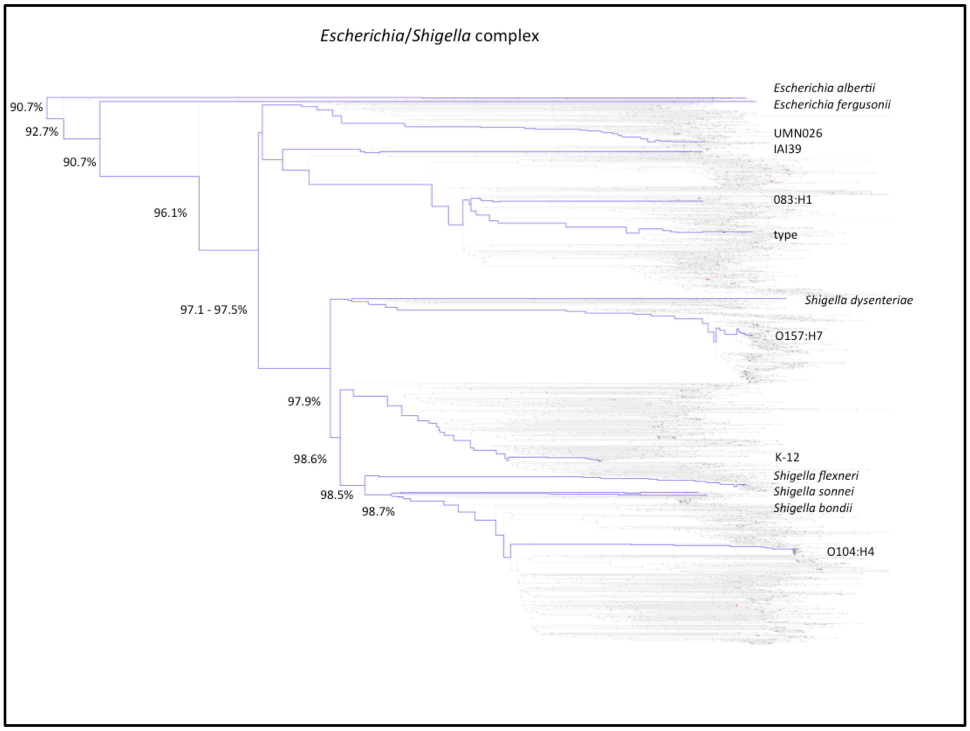

Some species are known to have taxonomic issues that can only be resolved in the literature. Escherichia coli contains all four species of Shigella, whose names have been conserved by a ruling of the Judicial Commission. This figure shows the 3000 genomes of E. coli in the kmer tree, together with Shigella and the sibling species E. fergusonii & E. albertii. The vast majority of coli genomes cluster within 97% ANI, with a small clade at 96.1% ANI. There is an ANI/kmer statistic anomaly at the top of the figure, highlighted on the next slide.

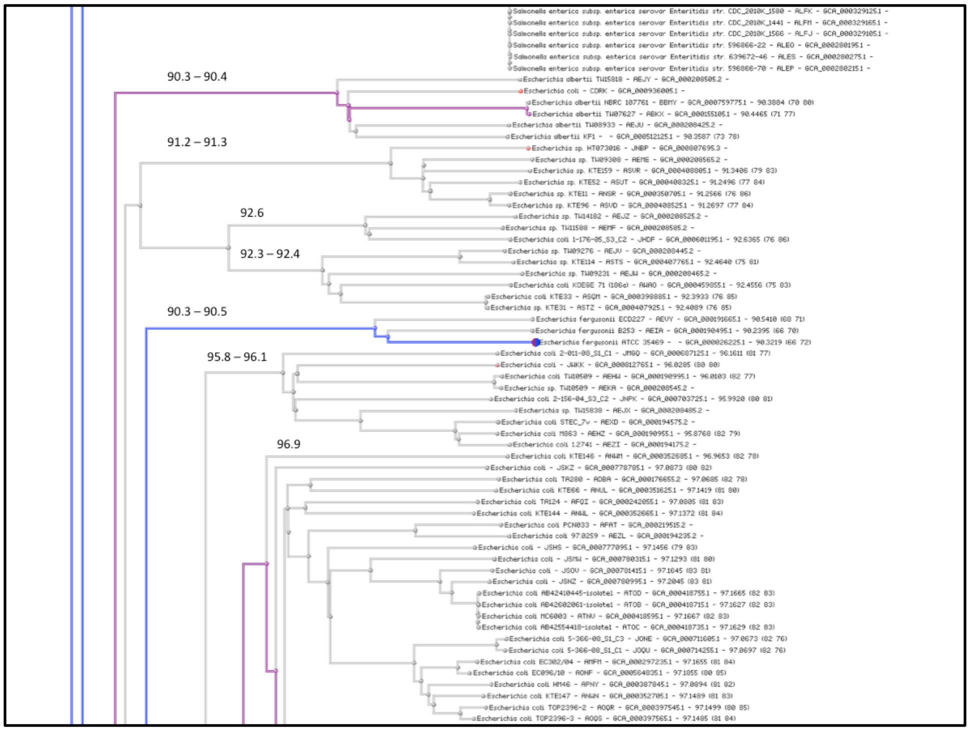

The clade of genomes between Escherichia albertii and Escherichia fergusonii in this figure show an ANI/kmer anomaly. The kmer scores place them further from Escherichia coli than the ANI scores do. Some as yet undetermined characteristic of these genomes affects the ANI & kmer statistics differently. This situation is rare.

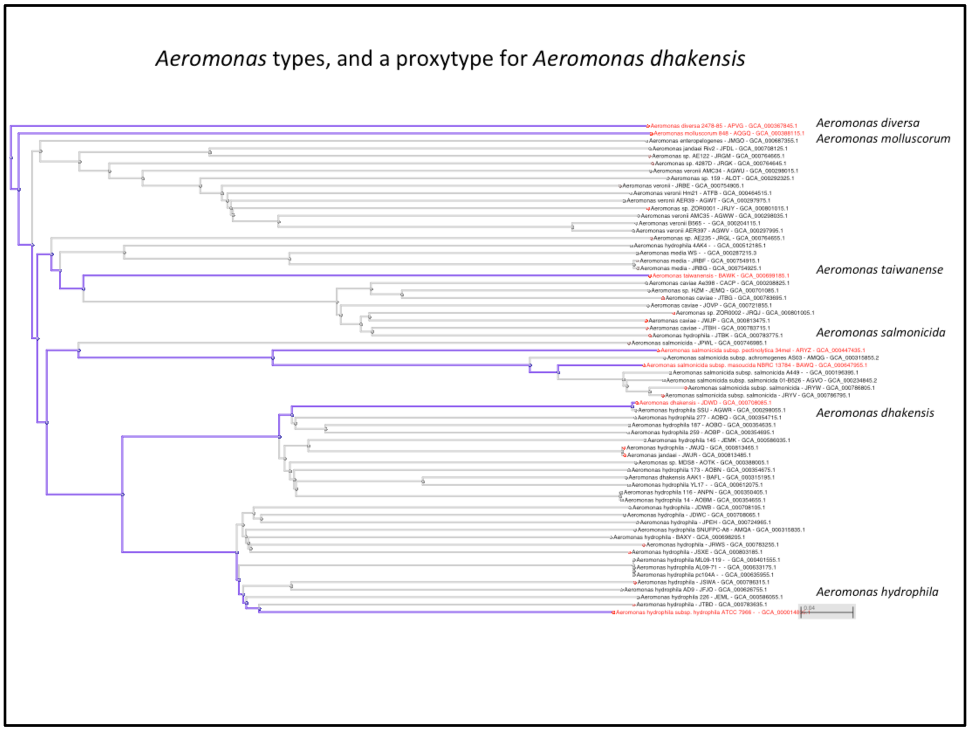

Aeromonas has become something of a test case for ANI-based genomic taxonomy in the literature. Here are the genomes from type for Aeromonas in GenBank as of 2014.

Aeromonas hydrophila

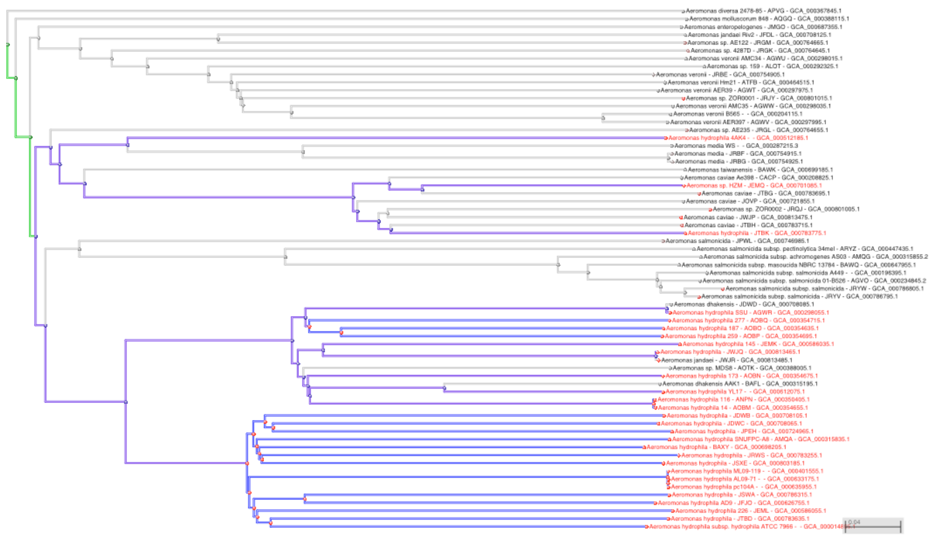

This is the kmer tree distribution of genomes submitted as Aeromonas hydrophila.

misidentified genomes in *Aeromonas*

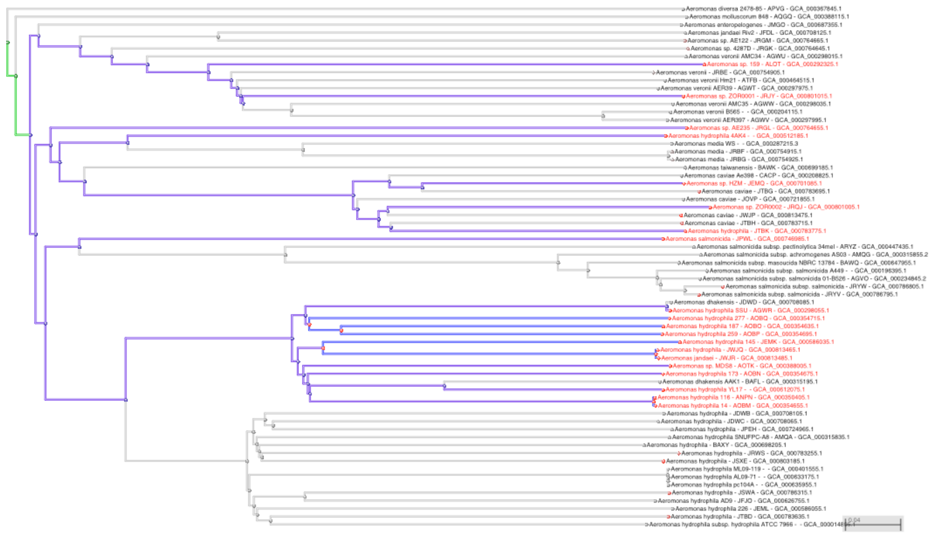

These are the Aeromonas genomes which would be given new taxonomic identifications based on the ANI analysis.
The large group at the bottom of the figure get new names due to the promotion of Aeromonas hydrophila subsp. dhakensis to species level.
(If those had been submitted with trinomials to begin with that would have happened automatically.)

type genomes in Aeromonas after Colston et al. 2014 (pmid 25406383)

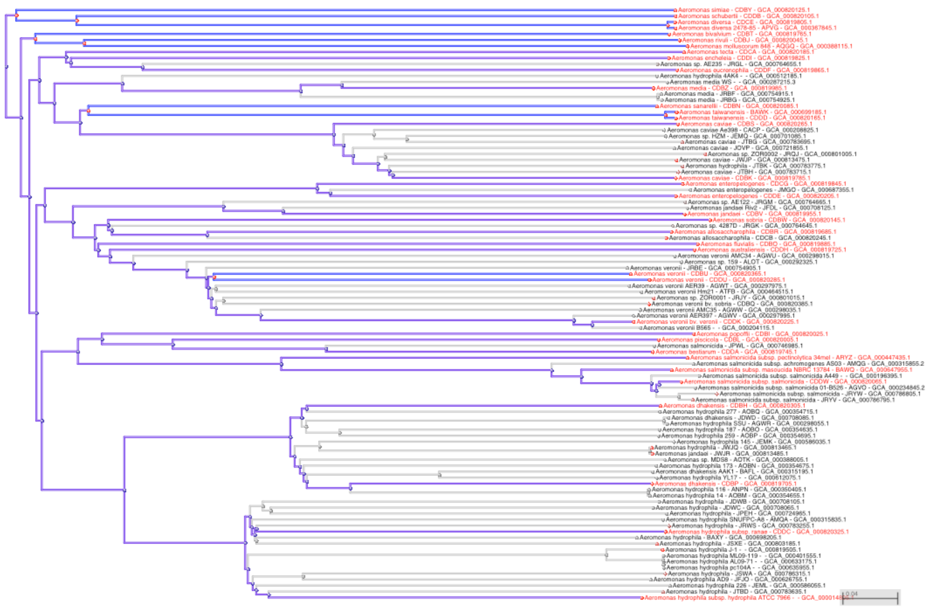

Colston et al. 2014 published the sequence of several dozen more type genomes in Aeromonas.

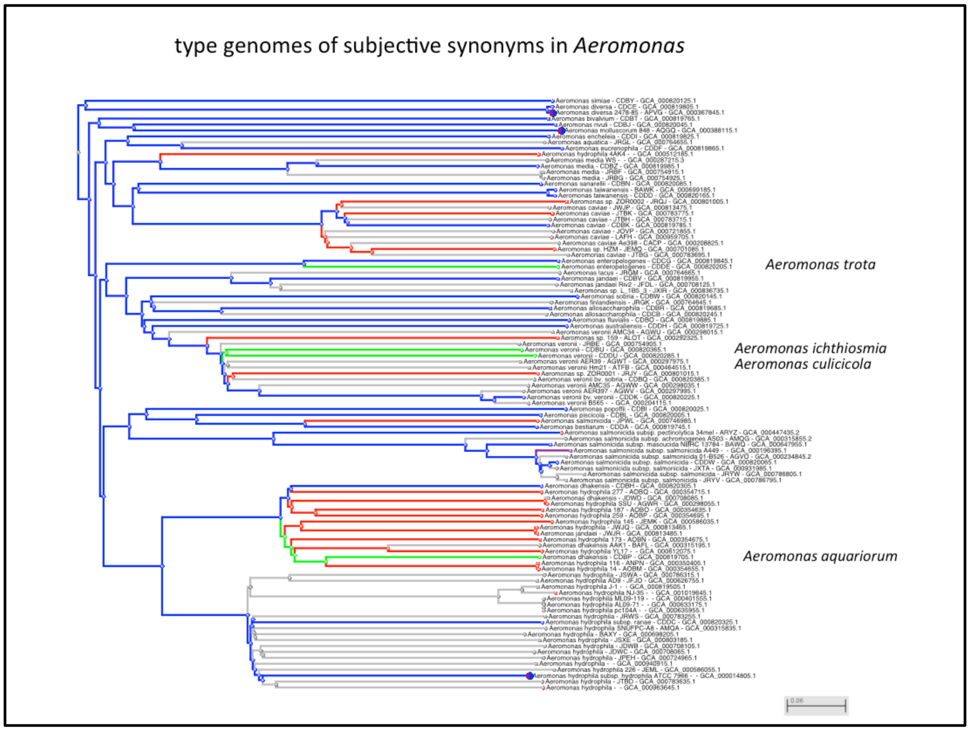

We flag type material from heterotypic synonyms separately from the primary type material. Traces leading to genomes from type strains of heterotypic synonyms are shown in green above. These placements in the kmer tree (and the corresponding ANI neighboring tables) confirm that it is reasonable to synonymize Aeromonas trota with Aeromonas enteropelogenes, to synonymize both Aeromonas ichthiosmia & Aeromonas culicicola with Aeromonas veronii,
and to synonymize Aeromonas aquariorum with Aeromonas dhakensis.

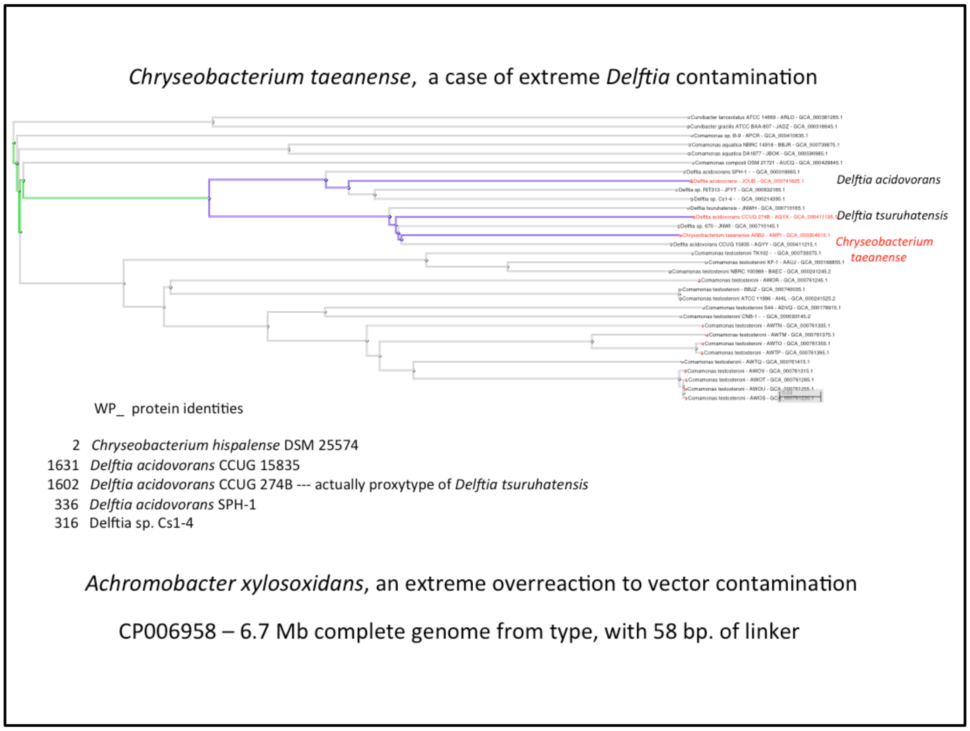

The elephant in the room here is contamination – we have never done a very good job of screening our bacterial genomes for contamination from other genomes. We identified several cases of massive contamination by investigating type genomes which appeared to be misplaced in the kmer tree. The putative type genome for Chryseobacterium taeanense had two proteins with sequences identical to Chryseobacterium hisapalense, but 3800 proteins with sequences identical to Delftia proteins. Turns out Delftia is a common contaminant of sequencing reagents. We currently have no type sequences from Delftia, but we can assign proxytype genomes for Delftia acidovorans & Delftia tsuruhatensis.
(The proxytype genome for Delftia tsuruhatensis was submitted as Delftia acidovorans, and would be renamed under the new system.) We have found several type genomes with enough contamination to misplace them in the kmer tree (as above, and with Thauera selenatis on slide 25). On the other extreme, a complete type genome was suppressed for 58 bp of linker when we could not get a response from the submitter. It has since been UNVERIFIED with a misc_feat comment on the linker contamination.

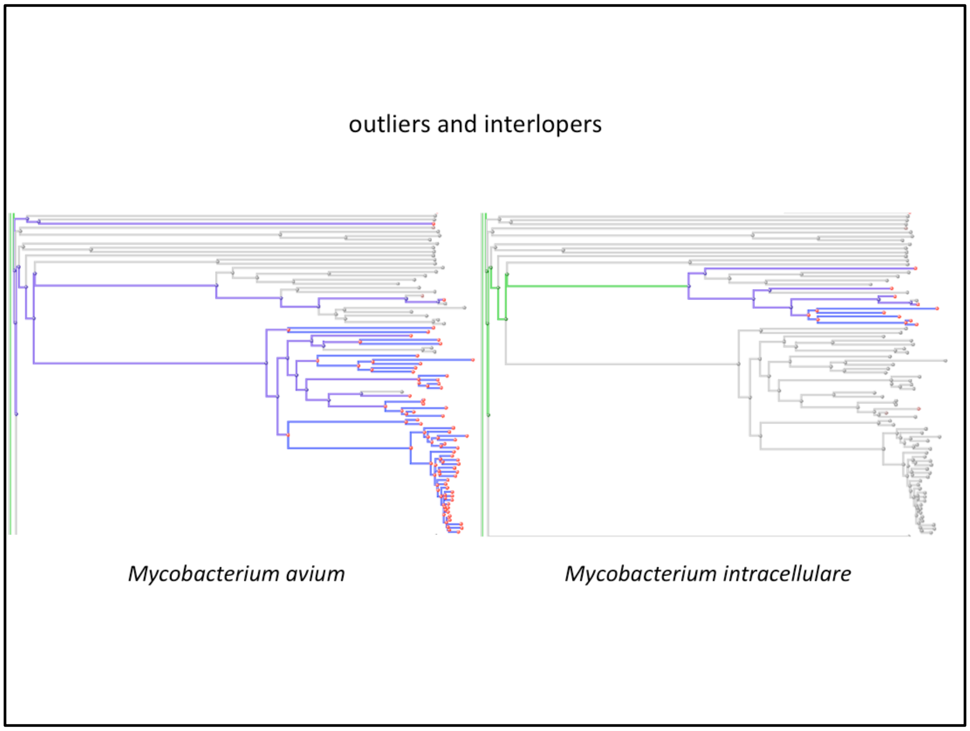

The Mycobacterium avium/intracellulare complex separates cleanly into clades in the kmer tree, with a few outliers & interlopers which may be misidentifications.

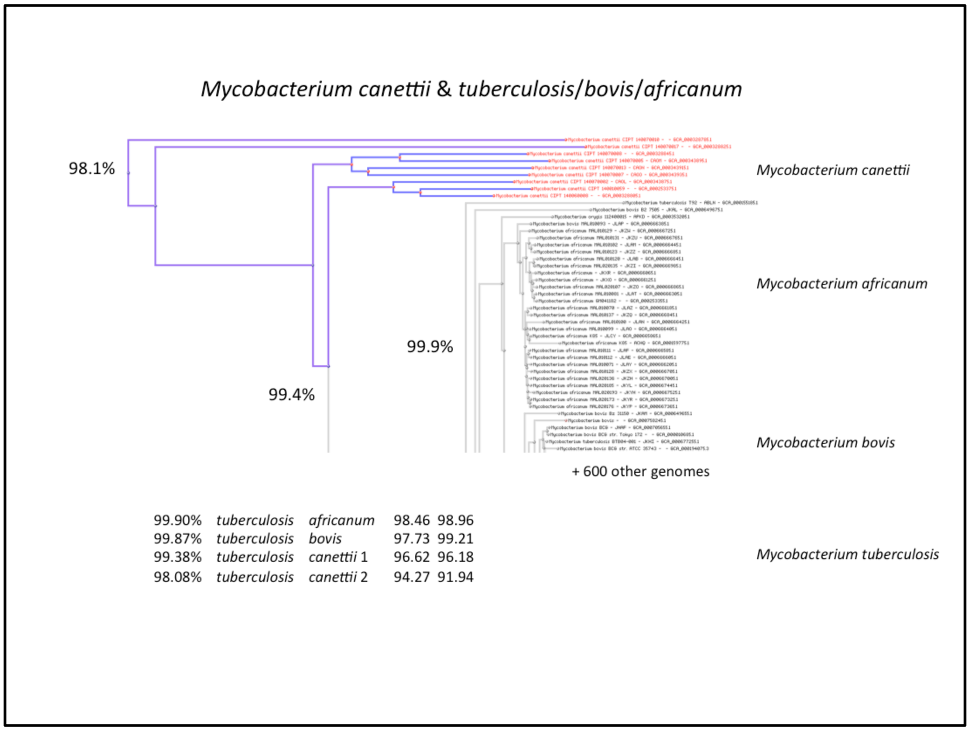

The Mycobacterium tuberculosis complex, on the other hand, is a know taxonomic problem case. Mycobacterium canettii is a paraphyletic species which is effectively but not validly published. Mycobacterium africanum, bovis & tuberculosis separate more or less cleanly into clades, but all of the genomes are within 99.9% ANI of each other. Thankfully, there are very few cases of species with serious taxonomic issues like this, and we can handle these as special cases.

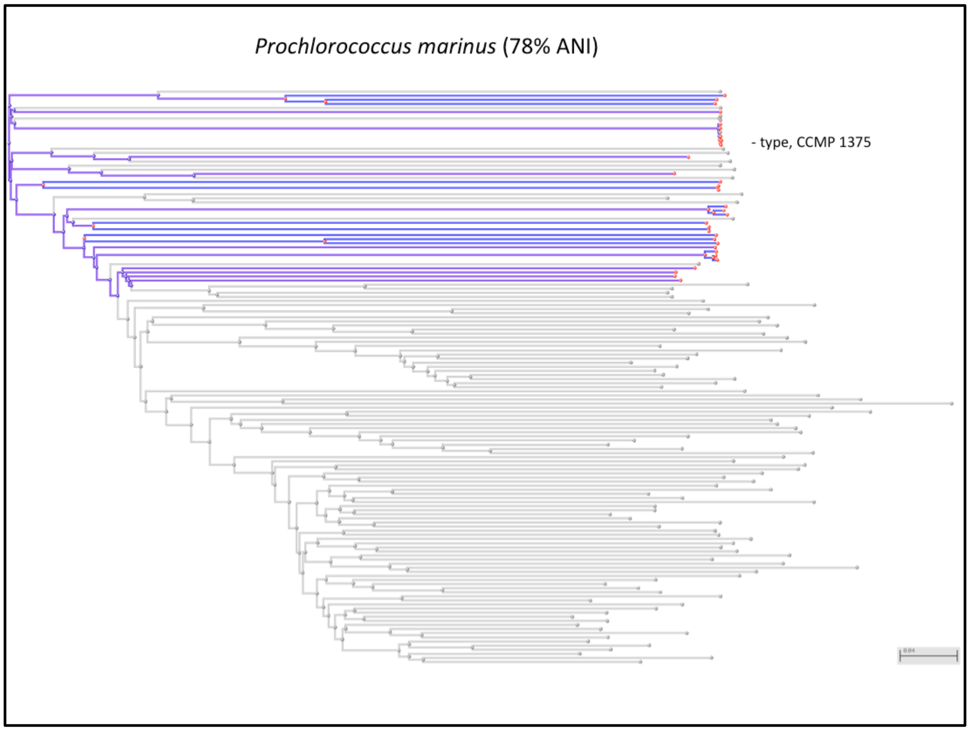

A few species span very very large ranges of ANI.

Candidatus Pelagibacter ubique

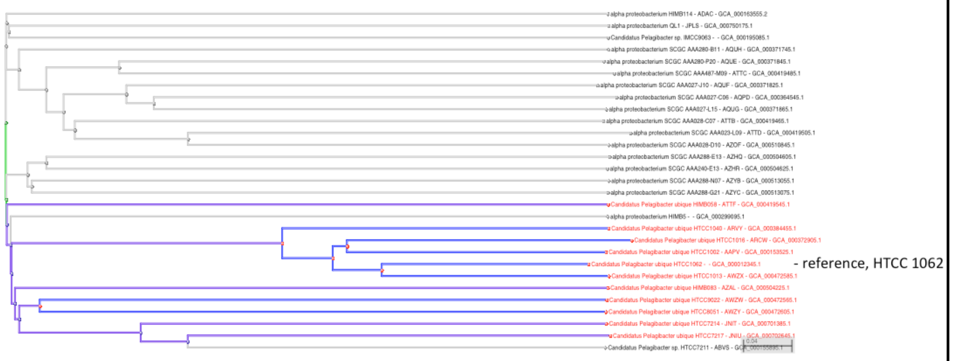

And another. Candidatus Pelagibacter ubique has not been validly published and therefore has no type strain, but we have designated HTCC 1062 as a ‘reference strain’. We can use this mechanism to name and cluster genomes derived from metagenomes and environmental samples.

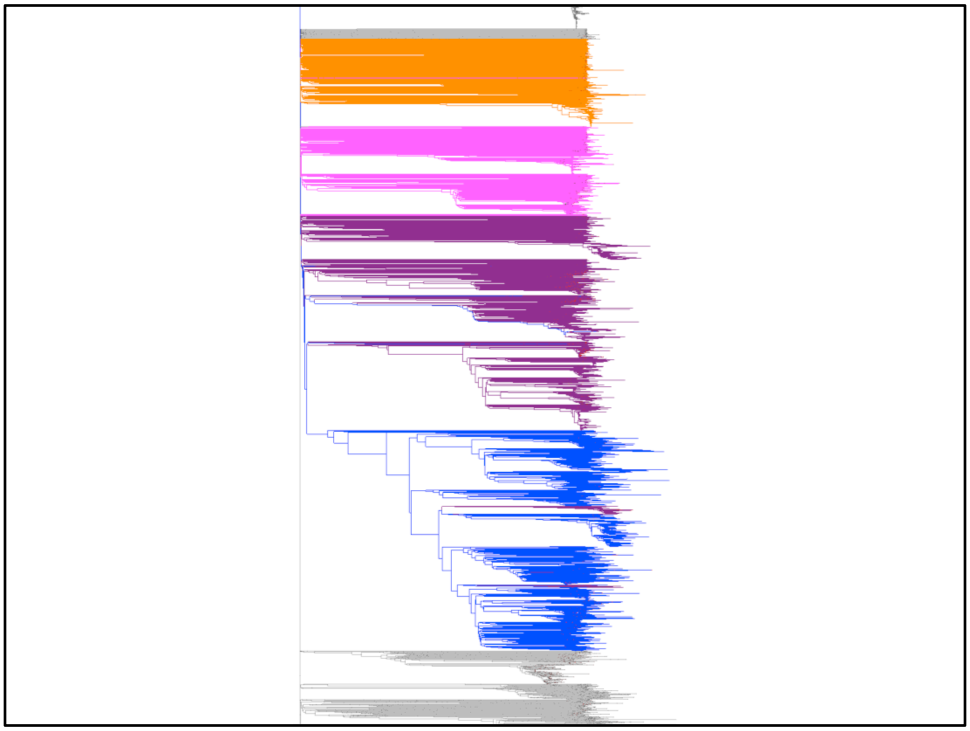

In the genome workbench it is easy to add metadata to the tree via attribute files. This tree shows the nested (and prioritized) queries genus=Escherichia > family=Enterobacteriaceae > class=Gammaproteobacteria > phylum=Proteobacteria. The purple patches nested within Escherichia are Shigella (aka STEC – shigatoxigenic Escherichia coli). The rest next nicely in spite of their placement near the spine of the tree, where we wouldn't expect to have much confidence in the topology.

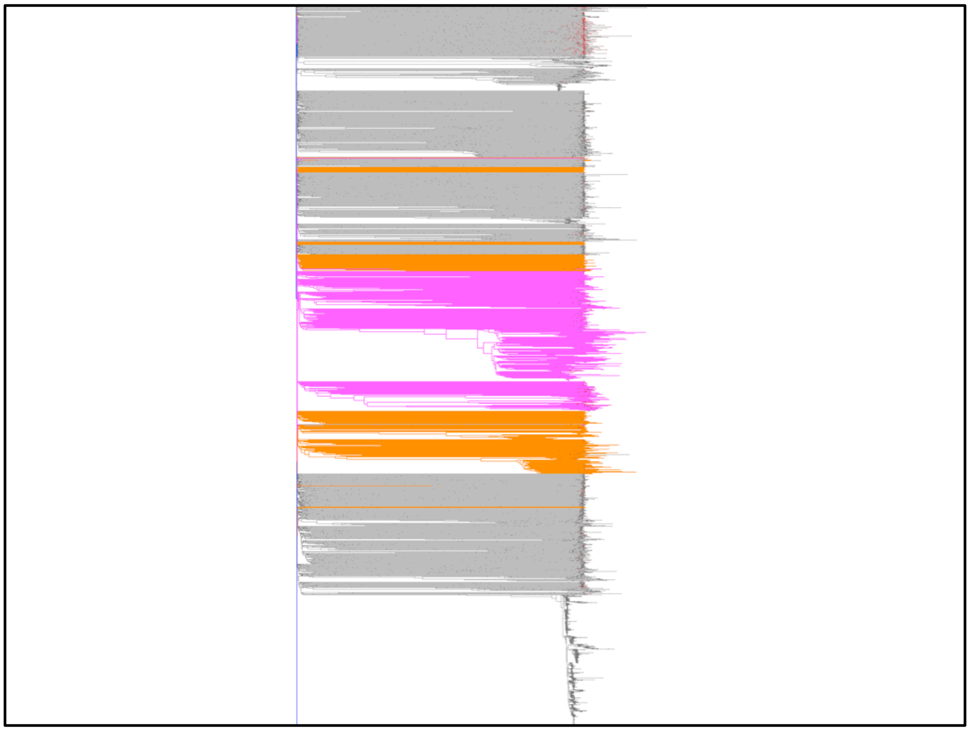

Other clades of gammaproteobacteria (pink) and proteobacteria (orange) map elsewhere with respect to the deep branches of the kmer tree.

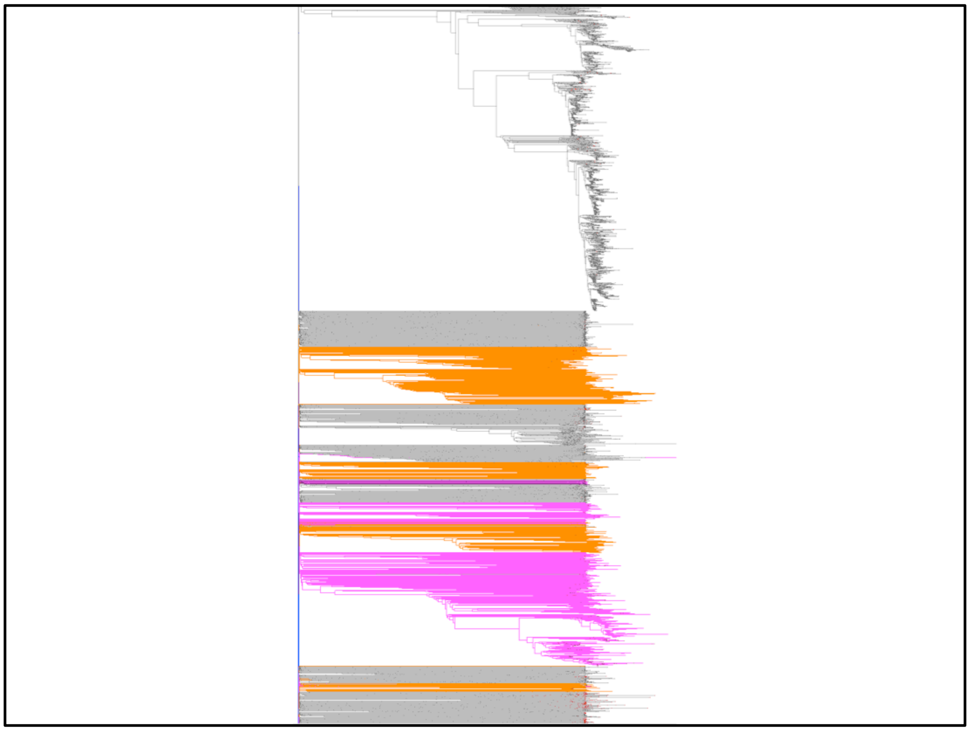

and this is the last of them.
Firmicutes above.
